# Identification of Conserved Amino Acid Substitutions During Serial Infection of Pregnant Cattle and Sheep With Bovine Viral Diarrhea Virus

**DOI:** 10.3389/fmicb.2018.01109

**Published:** 2018-06-06

**Authors:** Thibaud Kuca, Thomas Passler, Benjamin W. Newcomer, John D. Neill, Patricia K. Galik, Kay P. Riddell, Yijing Zhang, Paul H. Walz

**Affiliations:** ^1^Department of Clinical Sciences, College of Veterinary Medicine, Auburn University, Auburn, AL, United States; ^2^Ruminant Diseases and Immunology Research Unit, National Animal Disease Center, Agricultural Research Service, United States Department of Agriculture, Ames, IA, United States; ^3^Department of Pathobiology, College of Veterinary Medicine, Auburn University, Auburn, AL, United States

**Keywords:** bovine viral diarrhea virus, interspecies transmission, open reading frame, persistent infection, pestivirus, RNA virus, sheep, viral genetic diversity

## Abstract

Bovine viral diarrhea virus (BVDV) is an economically important pathogen of cattle that can also infect a wide range of domestic and wild species including sheep, goats, deer, camelids, and pigs. BVDV isolates are genetically highly diverse and previous work demonstrated that many substitutions were introduced in the viral genome during acute infections in cattle. In contrast, only limited information exists regarding changes occurring during BVDV infections in species other than cattle. The purpose of this study was to determine the changes introduced in the open reading frame (ORF) of the BVDV genome during serial infection of pregnant cattle and sheep with an isolate of bovine origin. Serial experimental inoculations were performed in six pregnant heifers and six pregnant ewes using BVDV-1b isolate AU526 in the first heifer and ewe, and serum from the preceding acutely infected dam thereafter. Complete ORF sequences were determined for 23 BVDV-1b isolates including AU526, one isolate from each pregnant dam, and one isolate from each BVDV-positive offspring born to these dams. Sequence comparison revealed that greater numbers of substitutions occurred during serial infection of pregnant sheep than of pregnant cattle. Furthermore, multiple host-specific amino acid changes were gradually introduced and conserved. These changes were more abundant in ovine isolates and occurred primarily in the E2 coding region. These results suggest that BVDV infections in heterologous species may serve as a significant source of viral genetic diversity and may be associated with adaptive changes.

## Introduction

Bovine viral diarrhea virus (BVDV) is an economically important pathogen of cattle worldwide, producing a wide range of clinical manifestations, including gastrointestinal, reproductive, and respiratory disease (Baker, 1995; Houe, 1999). BVDV is the prototypic member of the genus *Pestivirus* within the family *Flaviviridae*. Similar to other pestiviruses, BVDV has an enveloped, positive-sense, single-stranded RNA genome that is approximately 12.3 kb in length and contains a single open reading frame (ORF) encoding about 3,900 amino acids and two untranslated regions at the 5′ and 3′ ends (Collett et al., 1988a). Translation of the ORF generates a large polyprotein that is co- and post-translationally processed to yield four structural (C, E^rns^, E1, and E2) and eight non-structural (N^pro^, p7, NS2, NS3, NS4A, NS4B, NS5A, and NS5B) viral proteins (Collett et al., 1988b). BVDV isolates are genetically and antigenically highly diverse and can be segregated into two different species, BVDV-1 and BVDV-2 (Pellerin et al., 1994; Ridpath et al., 1994). Phylogenetic studies have demonstrated the existence of at least twenty-one BVDV-1 subgenotypes (1a-1u) and four BVDV-2 subgenotypes (2a-2d) (Giammarioli et al., 2015; Yesilbag et al., 2017). RNA viruses are characterized by high mutation rates and the lack of proofreading activity of RNA-dependent RNA polymerases is believed to be the main driving force for the generation of altered genomic sequences (Steinhauer et al., 1992; Sanjuán et al., 2010).

The clinical outcome of BVDV infection depends on various host and viral factors including reproductive status, gestational age, vaccination status, concurrent infections, viral virulence, and viral biotype (Baker, 1995). For instance, exposure of a naïve pregnant cow to BVDV causes an acute systemic infection that usually results in transplacental infection of the fetus (Gillespie et al., 1967). A remarkable feature of BVDV is the existence of two biotypes, cytopathic and non-cytopathic, according to whether or not the virus causes cell death in cultured cells (Lee and Gillespie, 1957; Underdahl et al., 1957). Although viral biotype does not correlate with virulence *in vivo*, the establishment of persistent infection is restricted to non-cytopathic viruses (Brownlie et al., 1989). When infection occurs in early pregnancy with a non-cytopathic virus, the fetal immune system may develop a highly specific immunotolerance resulting in lifelong viral persistence in this animal (Kendrick, 1971). Persistently infected (PI) animals shed large amounts of virus in most excretions and secretions, and therefore are the primary means of BVDV transmission in cattle populations (Coria and McClurkin, 1978; Houe, 1999).

Acute BVDV infections in pregnant cattle were found to result in 2.3 to 8 times more nucleotide substitutions than in non-pregnant cattle (Neill et al., 2012). Furthermore, most genetic changes introduced during the establishment of a persistent infection were shown to arise during the acute infection of the pregnant dam (Neill et al., 2012). BVDV infections in naïve pregnant cows are thus believed to be an important source of viral genetic diversity.

Although its natural host is cattle, BVDV can infect various species of domestic and wild ungulates. The establishment of persistent infection has been reported in seven species including alpaca, goats, sheep, swine, and white-tailed deer (Gard et al., 1976; Loken and Bjerkas, 1991; Terpstra and Wensvoort, 1997; Carman et al., 2005; Passler et al., 2007). It has been suggested that these species may serve as reservoirs for BVDV and as an additional source of viral diversity. Only limited information exists regarding genetic changes occurring during BVDV infections in species other than cattle. Multiple amino acid changes in the E2 coding region have been reported during the establishment of persistent infections in goats (Bachofen et al., 2013; Passler et al., 2014). Host-specific changes in the E2 and N^pro^ coding regions were also introduced during serial infection of pregnant cattle and sheep (Paton et al., 1997). Unfortunately, genetic analysis was often limited in these previous studies to the E2 coding region and parts of the N^pro^ and C coding regions of the viral genome.

The purpose of this study was to determine the changes introduced in the ORF of the BVDV genome during serial infection of pregnant cattle and sheep with an isolate of bovine origin. To infect a heterologous host, a virus must be able to efficiently bind a cell receptor, express its genes, replicate its genome, and produce infectious virions. However, there are several host barriers and defense mechanisms that may restrict virus infection including innate immune response and intercellular junctions (Kawai and Akira, 2006; Bergelson, 2009). One or more changes in the viral genome would be required to overcome each of these obstacles and thus we hypothesized that the number of viral genomic changes would be greater in sheep than in cattle during serial infection with a BVDV isolate of bovine origin.

## Materials and Methods

### Animals

All experimental procedures were performed with approval and under the guidelines of the Institutional Animal Care and Use Committee of Auburn University (Nos. 2015-2604 and 2015-2706). Six Angus-cross pregnant heifers were acquired from Auburn University Animal Health Research and six Suffolk-cross pregnant nulliparous ewes were acquired from a local commercial flock. The sheep experiment started in November 2014 and ended in May 2015, and the cattle experiment started in June 2015 and ended in September 2016. Prior to inclusion, animals were confirmed to be pregnant by transrectal or transabdominal ultrasonography, negative for BVDV by virus isolation (VI), and seronegative to BVDV by virus neutralization (VN). Animals were transported to the North Auburn BVDV unit at Auburn University and housed in isolated pens. At the time of inoculation, pregnant heifers and ewes were at 75 to 80 and 30 to 60 days of gestation, respectively. Animals were visually inspected daily for signs of illness and evidence of reproductive losses throughout the study period.

### Virus Inoculation and Sample Collection

On day 0, a physical examination was performed, pregnancy was confirmed by ultrasonography, and blood was collected for VI and VN prior to inoculation. Subsequently, the first heifer and the first ewe were each inoculated intravenously with approximately 1.0 × 10^6^ 50% tissue culture infective dose (TCID_50_) of BVDV-1b AU526. The non-cytopathic BVDV-1b AU526 virus had been isolated from the serum of a PI cow, which was part of a research herd at Auburn University Animal Health Research. This isolate was shown to cause persistent infections in goats and white-tailed deer (Passler et al., 2007, 2014). Virus stock had been passaged twice in Madin-Darby bovine kidney (MDBK) cells. Virus inoculum was prepared by adding 20 μl of virus stock at a titer of 5.0 × 10^7^ TCID_50_/ml to 980 μl of culture medium. Residual inoculum was stored at -80°C for estimation of received dose by virus titration.

On days 5 and 7 postinoculation (pi), a physical examination was performed and blood was collected for VI and quantitative reverse transcriptase PCR (qRT-PCR). Nasal swabs were also collected from the ewes for VI. The second, third, fourth, fifth, and sixth heifers were inoculated intravenously with 1 ml of BVDV-positive serum obtained on day 7 pi from the first, second, third, fourth, and fifth heifers, respectively. Analogously, the second ewe was inoculated intravenously with 1 ml of BVDV-positive serum obtained from the first ewe on day 7 pi. When serum samples obtained from pregnant ewes on days 5 and 7 pi were both positive for BVDV by qRT-PCR, the sample with the highest relative fluorescence unit value was chosen as inoculum. The third and sixth ewes were thus inoculated with serum obtained on day 5 pi from the second and fifth ewes, whereas the fourth and fifth ewes were inoculated with serum obtained on day 7 pi from the third and fourth ewes, respectively. In summary, serial experimental inoculations were performed in six pregnant heifers and six pregnant ewes using BVDV-1b isolate AU526 in the first heifer and ewe, and serum from the preceding acutely infected pregnant dam thereafter.

Pregnant dams were commingled following inoculation and pregnancies were allowed to proceed to term. Blood was collected every 28 days for VN until term. Ultrasound examinations were performed to assess pregnancy viability at the same intervals.

At the time of parturition or abortion, blood and skin biopsy samples were collected from offspring for VI and VN or antigen-capture enzyme-linked immunosorbent assay (ACE), respectively. Blood was also collected from dams for VN. Postmortem examinations were performed on aborted and stillborn fetuses as well as deceased offspring. Representative sections of placental and fetal tissues were collected for VI including liver, spleen, thymus, lymph nodes, kidney, lung, heart, gonad, small intestine, and brain. Additional blood samples were collected every 6 weeks from viable offspring for VI and VN until 6 months of age.

### Sample Processing

Blood collected in plain tubes was allowed to clot at room temperature for at least 30 min. Following centrifugation at 200 × *g* for 20 min, serum was harvested and stored at -80°C or immediately used in VI, VN, and qRT-PCR procedures. Blood collected in EDTA-containing tubes was processed to yield buffy coat. Following centrifugation at 700 × *g* for 30 min, buffy coat was removed using a sterile Pasteur pipette. Lysis of red blood cells was performed using 10 ml of 0.15 M ammonium chloride (NH_4_Cl). Buffy coat was washed in 10 ml of culture medium. Following centrifugation at 700 × *g* for 10 min, buffy coat was resuspended in 1 ml of culture medium to be used in VI procedures. Nasal swabs were placed in tubes containing culture medium to be used in VI procedures. Skin biopsy samples were immediately placed in tubes containing phosphate-buffered saline (PBS) to be used in ACE procedures. Sections of placental and fetal tissues were placed in stomacher bags containing 3 ml of culture medium and homogenized for 5 min with a Tekmar Stomacher^®^ laboratory blender (Tekmar Company, Cincinnati, OH, United States) to be used in VI procedures.

### Cells

Madin-Darby bovine kidney cells were purchased from the American Type Cell Culture Collection (CCL-22^TM^) and confirmed to be of bovine origin through amplification and sequencing of mitochondrial cytochrome c oxidase subunit 1 (CO1) gene (CellCheck^TM^, IDEXX Laboratories, Inc., Westbrook, ME, United States). PCR assays also demonstrated the absence of *Mycoplasma* and mammalian interspecies contamination (STAT-Myco^TM^, IDEXX Laboratories, Inc.). Cells were grown in minimum essential medium (MEM) with Earle’s salts supplemented with 10% equine serum, L-glutamine (0.02 mM), sodium bicarbonate (0.75 mg/ml), penicillin (100 U/ml), and streptomycin (100 μg/ml).

### Virus Isolation

Serum, buffy coat, and nasal swab samples as well as placental and fetal tissue samples were assayed for BVDV by passage through MDBK cells for 4 days. An immunoperoxidase monolayer assay (IPMA) was subsequently used to identify BVDV-positive cells.

Briefly, serum samples were assayed by adding 768 μl of serum to 192 μl of culture medium on 9.6-cm^2^ wells of a 6-well culture plate that had been seeded 24 h earlier with MDBK cells. The plates were incubated at 37°C for 1 h in humidified air containing 5% CO_2_. Subsequently, 3 ml of culture medium was added to each well. The plates were then incubated for 4 days. Following a single freeze-thaw cycle to release intracellular virus, cell lysates were assayed in triplicate by adding 10 μl of cell lysate to 90 μl of culture medium and 50 μl of culture medium containing MDBK cells on 0.32-cm^2^ wells of a 96-well culture plate. The plates were then incubated for 3 days. Following fixation, 50 μl of a mixture of two monoclonal antibodies, D89 and 20.10.6, was added to each well. These antibodies are specific for the envelope protein E2 and the non-structural protein NS3 of BVDV, respectively. The plates were incubated at 37°C for 1 h to allow antibody binding. Following washing with PBS containing 0.05% Tween 20 to remove unbound antibodies, 50 μl of diluted peroxidase-conjugated rabbit anti-mouse IgG was added to each well and culture plates were incubated at 37°C for 1 h. Following washing with PBS containing 0.05% Tween 20, 50 μl of aminoethyl carbazole substrate was added and culture plates were incubated at room temperature for 15 min. This enzyme substrate produces a reddish-brown color when oxidized by horseradish peroxidase. Color change was examined by use of light microscopy and compared with that of positive- and negative-control samples included on each culture plate.

Buffy coat and nasal swab samples as well as placental and fetal tissue samples were assayed by adding 1 ml of sample suspension on 9.6-cm^2^ wells of a 6-well culture plate that had been seeded 24 h earlier with MDBK cells. The plates were processed as described above and cell lysates were subsequently assayed for BVDV in triplicate using an IPMA as described above.

### Virus Titration

Virus titration was performed on the initial inoculum, VI-positive serum samples, and VI-positive fetal tissue samples. The quantity of BVDV was determined by multiple 10-fold dilutions of samples using the statistical method of Reed and Muench (1938). Briefly, each sample was assayed by adding 10 μl of sample to 90 μl of culture medium on each of three 0.32-cm^2^ wells of a 96-well culture plate that had been seeded 24 h earlier with MDBK cells. Serial 10-fold dilutions were made in culture medium, retaining 90 μl of each dilution per well. The plates were incubated at 37°C for 3 days in humidified air containing 5% CO_2_. The IPMA described above was subsequently used to identify BVDV-positive cells.

### Virus Neutralization

A standard VN microtiter assay was used to detect and quantify neutralizing antibodies to BVDV-1b AU526 in serum samples obtained from dams and their offspring. Serum samples were heat inactivated by incubation at 56°C for 30 min. Each sample was assayed in triplicate by adding 50 μl of sample to 50 μl of culture medium on each of three 0.32-cm^2^ wells of a 96-well culture plate that had been seeded 24 h earlier with MDBK cells. Serial twofold dilutions were made in culture medium, retaining 50 μl of each dilution per well. An equal volume of culture medium containing 200 TCID_50_ of BVDV-1b AU526 was added to each well. The plates were incubated at 37°C for 1 h in humidified air containing 5% CO_2_ and 50 μl of culture medium containing MDBK cells was then added to each well. The plates were incubated for 3 days and subsequently assayed for BVDV using the IPMA described above. Antibody titer was defined as the reciprocal of the highest dilution at which 2 out of 3 wells were free of staining.

### Antigen-Capture Enzyme-Linked Immunosorbent Assay

Skin biopsy samples obtained from calves were assayed for BVDV by an independent laboratory using the BVDV PI X2 Test kit (IDEXX Laboratories, Inc.) according to the manufacturer’s instructions. This test relies on monoclonal antibodies directed against the protein E^rns^ of BVDV to capture the viral antigen and detects antigen-antibody complexes with enzyme-conjugated antibody by spectrophotometry. The sample to positive (S/P) ratio was calculated for each sample and a ratio of 0.3 or greater was considered positive.

Skin biopsy samples obtained from lambs were assayed for BVDV using the SNAP^®^ BVDV Antigen Test kit (IDEXX Laboratories, Inc.) according to the manufacturer’s instructions. This assay uses the same monoclonal antibodies against the BVDV protein E^rns^.

### Quantitative Reverse Transcriptase PCR

Serum samples obtained from pregnant ewes on days 5 and 7 pi were assayed for BVDV by qRT-PCR. Viral RNA was isolated from serum samples using the High Pure PCR Template Preparation Kit (Roche Diagnostics Corporation, Indianapolis, IN, United States). Briefly, 200 μl of serum was added to 40 μl of proteinase K with 200 μl of binding buffer into a 1.5-ml microcentrifuge tube. Following incubation at 70°C for 10 min, 100 μl of isopropanol was added and the solution was centrifuged through a filter tube containing glass fibers for 1 min at 11,000 × *g*. Following centrifugation, 500 μl of inhibitor removal buffer was added and the filter tube was subsequently centrifuged at 11,000 × *g* for 1 min. Following centrifugation, the filter tube was washed twice with 450 μl of wash buffer and centrifuged at 11,000 × *g* for 1 min. Viral RNA on the glass fibers was then eluted in 60 μl of elution buffer by centrifuging at 8,000 × *g* for 1 min.

The qRT-PCR assay was performed using the iTaq^TM^ Universal SYBR^®^ Green One-Step kit (Bio-Rad Laboratories, Inc., Hercules, CA, United States) on the Bio-Rad CFX96 Touch^TM^ real-time PCR instrument (Bio-Rad Laboratories, Inc.). Briefly, 5 μl of viral RNA was added on the wells of a PCR plate containing 10 μl of iTaq^TM^ Universal SYBR^®^ Green Supermix, 0.25 μl of iScript^TM^ reverse transcriptase, 0.3 μl of each forward and reverse primer (20 μM) and 4.15 μl of ultra-pure water. The PCR primers BVDV_F (5′-TAGCCATGCCCTTAGTAGGAC-3′) and BVDV_R (5′-GACGACTACCCTGTACTCAGG-3′) were amplifying a 290-bp sequence of the 5′ untranslated region of the BVDV genome. Thermal cycling protocol included 10 min of reverse transcription at 50°C and 1 min of polymerase activation and DNA denaturation at 95°C followed by 40 cycles of amplification with denaturation at 95°C for 10 s and primer annealing and extension at 56.5°C for 30 s. Following the final amplification cycle, a melting curve was obtained by cooling to 65°C and then slowly heating to 95°C at 0.1°C/s while continuously monitoring fluorescence. Data were analyzed using the Bio-Rad CFX Manager software (Bio-Rad Laboratories, Inc.).

### Viral Genome Sequencing

VI-positive serum, buffy coat, and fetal tissue samples containing at least 10^4^ TCID_50_/ml of virus were directly used in genome sequencing procedures. VI-positive samples with a lower virus titer were propagated in MDBK cells. Growth in cell culture was limited to one passage to minimize the introduction of artifactual changes in the viral genome. Virus was harvested by freezing and thawing the infected cells and samples were stored at -80°C.

Sequencing of BVDV isolates was performed as previously described (Neill et al., 2014). A random primed, barcoded library technique utilizing primers composed of 20 bases of known sequence with 8 random bases at the 3′ end was used. These 28-mer primers conferred sequence independence by random priming both first and second strand cDNA synthesis.

To adapt sequencing to the MiSeq^TM^ platform (Illumina, Inc., San Diego, CA, United States), double-stranded cDNA PCR reactions were transferred to 96-well culture plates and the DNA was size-fractionated and purified using paramagnetic beads (Agencourt^®^ AMPure^®^ XP, Beckman Coulter, Indianapolis, IN, United States) at a DNA-to-bead ratio of 1:0.8. This resulted in the removal of DNA fragments of less than 300 bp including adaptor dimers and unligated adaptors. The DNA was prepared for sequencing using the Nextera^TM^ DNA Library Preparation Kit (Illumina, Inc.) according to the manufacturer’s instructions. The use of indexed barcoded PCR primers allowed multiplexing of libraries. The DNA was subjected to sequence analysis with the MiSeq^TM^ platform using the MiSeq Reagent kit v2 (Illumina, Inc.) for 2 × 150 base paired-end sequencing.

Genomic sequences were assembled and edited using the Lasergene SeqMan NGen software (DNASTAR, Inc., Madison, WI, United States) and the genomic sequence of AU526 as the assembly reference. Sequences were further edited using the CodonCode Aligner software (Codoncode Corporation, Centerville, MA, United States). Numbering of nucleotides started at the ATG initiation codon of the ORF. Isolates were named using the animal’s identification number. Dams were identified using a letter for the species (B, bovine; O, ovine) and the order number in the inoculation series. Offspring were identified using the dam’s identification number and a letter for the birth order (A, first; B, second).

### Data Analysis

Pairwise comparisons of genomic sequences were performed using the MEGA software (version 7.0.18^1^). Ambiguous nucleotides were not considered to be substitutions and thus not included in the results of pairwise comparisons. Statistical significance of the difference between the median number of substitutions was evaluated using the Wilcoxon rank sum test. The number of positions affected by substitutions was compared using the Pearson’s Chi-squared test. The number of substitutions in isolates from offspring that were not detected in isolates from their dams was also compared using the Pearson’s Chi-squared test. The ratio of the number of observed substitutions to the number of expected substitutions assuming a random distribution of substitutions across the viral genome was calculated for each protein-coding region.

### Accession Numbers

The viral genomic sequences determined in this study have been deposited in GenBank under accession numbers MG950344 to MG950366 and MH311874 to MH311881.

## Results

### Clinical, Virological, and Serological Findings

All heifers and ewes were confirmed to be seronegative to BVDV and free of BVDV and clinical disease prior to inoculation (**Table 1**). Virus titration of residual inoculum demonstrated that the viral dose received was 6.2 × 10^5^ TCID_50_ for the first heifer and 2.0 × 10^6^ TCID_50_ for the first ewe. Infection was confirmed in all dams by positive VI results on day 5 or 7 pi and seroconversion by day 28 pi (**Table 1**). Clinical signs of disease were not observed in any of the heifers nor in two ewes (O3 and O6). In the remaining ewes, inappetence, lethargy, and rectal temperature greater than 39.5°C were noted at least once during the first week pi.

**Table 1:** Virological and serological analysis of serial inoculation of pregnant heifers and ewes with BVDV.

		0 dpi		5 dpi	7 dpi	28 dpi	56 dpi	84 dpi	112 dpi	140 dpi	168 dpi	Day of parturition or abortion		
Dam	Fetus*^a^*	VN	VI*^b^*	VI*^c,d^*	VI*^c,e^*	VN	VN	VN	VN	VN	VN	VN	Dpi	Offspring
B1	80	<2	-/-	+	+/+	256	1,024	4,096	4,096	8,192	8,192	2,048	197	Stillborn BVDV+ calf
B2	80	<2	-/-	-	+/+	1,024	2,048	4,096	4,096	8,192	8,192	4,096	194	Live viable BVDV+ calf
B3	75	<2	-/-	+	+/+	512	2,048	2,048	4,096	4,096	8,192	8,192	197	Live viable BVDV+ calf
B4	80	<2	-/-	+	-/+	1,024	4,096	8,192	4,096	8,192	8,192	4,096	199	Live viable BVDV+ calf
B5	80	<2	-/-	+	-/+	1,024	1,024	4,096	8,192	8,192	8,192	4,096	192	Live viable BVDV+ calf
B6	80	<2	-/-	-	-/+	1,024	1,024	2,048	2,048	4,096	8,192	32,768	207	Live viable BVDV+ calf
														
O1	50	<2	-/-	+/+/NT	-/+/NT	512	16,384	32,768	NA	NA	NA	32,768	96	Live non-viable BVDV+ lamb/mummified fetus
O2	40	<2	-/-	+/+/NT	+/+/+	256	2,048	2,048	NA	NA	NA	8,192	106	Two stillborn BVDV+ lambs
O3	60	<2	-/-	+/+/-	+/+/+	2,048	2,048	NA	NA	NA	NA	1,024	77	Live non-viable BVDV- lamb
O4	40	<2	-/-	+/+/-	+/+/-	1,024	16,384	16,384	NA	NA	NA	16,384	102	Live non-viable BVDV+ lamb
O5	35	<2	-/-	+/+/+	+/+/+	256	NA	NA	NA	NA	NA	512	46	Uterine contents not recovered
O6	30	<2	-/-	+/+/-	+/+/-	512	512	NA	NA	NA	NA	8,192	70	BVDV- aborted fetus

^a^*Estimated gestational age at the time of inoculation based on ultrasound findings. *^*b*^*Results are given for serum samples followed by those for buffy coat samples. *^*c*^*Results are given for serum samples followed by those for buffy coat and nasal swab samples. *^*d*^*Only serum samples from heifers were tested at 5 dpi. *^*e*^*Only serum and buffy coat samples from heifers were tested at 7 dpi. dpi, day postinoculation; NA, not applicable; NT, not tested; VI, virus isolation; VN, virus neutralization; +, positive; -, negative*.

All heifers carried the pregnancy to term and delivered one stillborn calf and five live calves that were viable and nursed shortly after birth (**Table 1**). Persistent infection with BVDV was demonstrated in these five calves by repeated VI from blood samples obtained at 21, 42, 84, and 168 days of age (**Table 2**). The stillborn calf (B1A) was of normal size and appearance. Transplacental infection with BVDV was confirmed in this calf by VI from a composite of tissue samples including liver, spleen, thymus, kidney, lung, and heart (**Table 2**).

**Table 2:** Virological and serological analysis of calves born to heifers infected with BVDV in early pregnancy.

	Day of birth					21 doa			42 doa			84 doa			168 doa		
	Serum		BC	Skin	Tissues*^a^*	Serum		BC	Serum		BC	Serum		BC	Serum		BC
Offspring	VN	VI	VI	ACE	VI	VN	VI	VI	VN	VI	VI	VN	VI	VI	VN	VI	VI
B1A	<2	-	NA	-	+	NA	NA	NA	NA	NA	NA	NA	NA	NA	NA	NA	NA
B2A	32,768	-	-	+	NA	2,048	-	+	8	-	+	4	+	+	<2	+	+
B3A	<2	+	+	+	NA	4,096	-	+	4	-	+	4	-	+	8	-	+
B4A	32,768	-	+	-	NA	4,096	-	+	<2	+	+	<2	+	+	<2	+	+
B5A	65,536	-	-	+	NA	128	-	+	<2	+	+	<2	+	+	<2	+	+
B6A	32,768	-	-	-	NA	16,384	-	-	8	+	+	<2	+	+	<2	+	+

^a^*Composite tissue including liver, spleen, thymus, kidney, lung, and heart. ACE, antigen-capture ELISA; BC, buffy coat; doa, days of age; NA, not applicable; VI, virus isolation; VN, virus neutralization; +, positive; -, negative*.

Two ewes (O5 and O6) aborted on days 46 and 70 pi at approximately 80 and 100 days of gestation, respectively (**Table 1**). The aborted fetus (O6A) had brachygnathia superior. Uterine contents were not recovered from the other ewe. The remaining ewes gave birth to three live non-viable lambs, two stillborn lambs, and one mummified fetus (**Table 1**). Two live lambs (O1A and O3A) were weak, underweight, and unable to stand or nurse at birth. These lambs were fed colostrum by oroesophageal tubing, but were ultimately euthanized within 24 h of birth. Lamb O1A had brachygnathia inferior and postmortem examination revealed thymic atrophy in this lamb. The third live lamb (O4A) was observed to nurse after birth. However, its condition deteriorated rapidly and it was ultimately euthanized 24 h after birth. The stillborn lambs (O2A and O2B) were of normal size and appearance. The mummified fetus (O1B) was 39.5-cm long and fetal death was estimated to have occurred 70 days pi at 120 days of gestation.

Transplacental infection with BVDV was confirmed in two non-viable lambs and two stillborn lambs by VI from blood and tissue samples and by detection of BVDV antigen in skin biopsy samples (**Table 3**). The presence of live virus and the absence of precolostral antibodies to BVDV suggested that these lambs were born PI with BVDV. Transplacental infection was confirmed in the third non-viable lamb (O3A) by a high antibody titer in precolostral serum. Virus was not recovered from this lamb.

**Table 3:** Virological and serological analysis of lambs born to ewes infected with BVDV in early pregnancy.

		Day of birth						
		Serum		BC	Skin	Tissues*^b^*	Brain	Spleen
Offspring	Fetus*^a^*	VN	VI	VI	ACE	VI	VI	VI
O1A	50	<2	+	+	+	-	+	+
O1B	50	NA	NA	NA	-	-	NT	NT
O2A	40	NT	NT	NT	+	+	NT	NT
O2B	40	NT	NT	NT	+	+	NT	NT
O3A	65	8,192	-	-	-	-	NT	NT
O4A	40	<2	+	+	+	+	NT	NT
O6A	30	NT	NT	NT	-	-	NT	NT

^a^*Estimated gestational age at the time of inoculation based on ultrasound findings. *^*b*^*Composite tissue including liver, spleen, thymus, kidney, lung, and heart. ACE, antigen-capture ELISA; BC, buffy coat; NA, not applicable; NT, not tested; VI, virus isolation; VN, virus neutralization; +, positive; -, negative*.

### Genomic Sequence Analysis

Complete ORF sequences were determined for 23 BVDV-1b isolates including AU526, one isolate from each acutely infected dam, and one isolate from each VI-positive offspring born to these dams. For all but one dam, serum samples obtained on day 5 or 7 pi were used in genome sequencing procedures. A buffy coat sample obtained on day 7 pi was used for the sixth heifer (B6). Serum or tissue samples were used for offspring. The first VI-positive serum sample collected after birth was used for PI calves. Five sequences contained ambiguous nucleotides with a median of 1 nucleotide (range, 1–2).

When compared to AU526, a median of 23 nucleotide substitutions (range, 9–26) was observed in isolates from acutely infected pregnant heifers (**Table 4**). In contrast, there was a median of 46 nucleotide differences (range, 0–51) between AU526 and isolates from pregnant ewes. Similar to their dams, a median of 27 substitutions (range, 23–34) was observed in isolates from calves and a median of 50 substitutions (range, 49–52) was observed in isolates from lambs. The difference between the median number of nucleotide changes observed in bovine and ovine isolates was statistically significant (*P* = 0.002).

**Table 4:** Nucleotide substitutions between AU526 and isolates from acutely infected dams and their offspring.

	Total	Transitions	Transversions	Ratio	N^pro^	C	E^rns^	E1	E2	p7	NS2	NS3	NS4A	NS4B	NS5A	NS5B	% S*^a^*	% NS*^b^*
**AU526:Dam**																		
AU526:B1	9	8	1	8.0	0	0	1	0	1	0	2	2	0	1	1	1	22.2	77.8
AU526:B2	16	11	5	2.2	2	0	1	0	3	0	1	4	0	2	0	3	25.0	75.0
AU526:B3	25	20	5	4.0	2	1	0	0	4	1	0	3	1	5	5	3	20.0	80.0
AU526:B4	26	21	5	4.2	2	1	1	1	5	1	0	4	1	2	5	3	30.8	69.2
AU526:B5	25	23	2	11.5	1	0	1	0	4	0	2	2	0	4	7	4	20.0	80.0
AU526:B6	20	17	3	5.7	2	1	0	0	4	1	0	2	1	1	5	3	25.0	75.0
Median	23	19	4	4.9	2	1	1	0	4	1	1	3	1	2	5	3	23.6	76.4
Mean	20	17	4	5.9	2	1	1	0	4	1	1	3	1	3	4	3	23.8	76.2
AU526:O1	0	0	0		0	0	0	0	0	0	0	0	0	0	0	0		
AU526:O2	37	28	9	3.1	0	1	6	3	3	0	3	9	0	2	2	8	35.1	64.9
AU526:O3	42	33	9	3.7	1	2	4	2	6	0	4	9	0	3	2	9	33.3	66.7
AU526:O4	49	40	9	4.4	1	3	7	3	6	0	4	9	0	3	2	11	38.8	61.2
AU526:O5	49	40	9	4.4	1	3	7	3	6	0	4	9	0	3	2	11	38.8	61.2
AU526:O6	51	42	9	4.7	1	3	7	3	7	0	4	9	0	3	2	12	39.2	60.8
Median	46	37	9	4.4	1	3	7	3	6	0	4	9	0	3	2	10	38.8	61.2
Mean	38	31	8	4.1	1	2	5	2	5	0	3	8	0	2	2	9	37.0	63.0
**AU526:Offspring**								
AU526:B1A	34	30	4	7.5	0	2	3	4	4	0	4	6	0	5	0	4	69.2	30.8
AU526:B2A	26	21	5	4.2	2	1	0	0	6	1	0	4	1	1	6	4	42.9	57.1
AU526:B3A	23	18	5	3.6	2	1	0	1	4	1	0	4	1	1	5	3	50.0	50.0
AU526:B4A	28	24	4	6.0	2	1	0	1	9	1	0	4	1	1	5	3	70.0	30.0
AU526:B5A	25	21	4	5.3	2	1	0	2	4	1	0	4	1	2	5	3	50.0	50.0
AU526:B6A	27	22	5	4.4	2	1	0	0	5	1	0	6	1	1	6	4	28.6	71.4
Median	27	22	5	4.8	2	1	0	1	5	1	0	4	1	1	5	4	50.0	50.0
Mean	27	23	5	5.2	2	1	1	1	5	1	1	5	1	2	5	4	51.8	48.2
AU526:O1A	49	35	14	2.5	2	1	3	5	3	2	4	13	1	3	4	8	24.5	75.5
AU526:O2A	49	40	9	4.4	1	3	7	3	6	1	4	9	0	3	2	10	38.8	61.2
AU526:O2B	51	42	9	4.7	1	4	8	3	5	1	4	9	0	3	2	11	39.2	60.8
AU526:O4A	52	43	9	4.8	1	3	7	3	7	1	4	9	0	3	2	12	38.5	61.5
Median	50	41	9	4.6	1	3	7	3	6	1	4	9	0	3	2	11	38.6	61.4
Mean	50	40	10	4.1	1	3	6	4	5	1	4	10	0	3	3	10	35.2	64.8

^a^*Percentage of substitutions within structural protein-coding regions. *^*b*^*Percentage of substitutions within non-structural protein-coding regions.*

Most substitutions were transitions with a median transition-to-transversion ratio of 4.9 in isolates from pregnant heifers and 4.4 in isolates from pregnant ewes (**Table 4**). A median transition-to-transversion ratio of 4.8 and 4.6 was observed in isolates from calves and lambs, respectively. In both groups of isolates, substitutions were more prevalent in non-structural protein-coding regions with 15 and 18% of changes in bovine isolates occurring in the NS3 and NS5A coding regions, respectively. Similarly, 20 and 21% of changes in ovine isolates occurred in the NS3 and NS5B coding regions, respectively. However, 18% of changes in bovine isolates were also found in the E2 coding region and 13% of changes in ovine isolates were also found in the E^rns^ coding region. Analysis of the number of changes per protein-coding region revealed that nucleotide substitutions were essentially randomly distributed (**Table 5**).

**Table 5:** Ratio of the number of observed nucleotide substitutions per protein-coding region to the number of expected substitutions assuming a random distribution across the viral genome.

	Total	N^pro^	C	E^rns^	E1	E2	p7	NS2	NS3	NS4A	NS4B	NS5A	NS5B
**AU526:Dam**													
AU526:B1	9	0.00	0.00	1.91	0.00	1.16	0.00	2.14	1.17	0.00	1.25	0.87	0.60
AU526:B2	16	2.90	0.00	1.07	0.00	1.95	0.00	0.60	1.32	0.00	1.40	0.00	1.02
AU526:B3	25	1.86	1.53	0.00	0.00	1.67	2.56	0.00	0.63	2.44	2.25	1.57	0.65
AU526:B4	26	1.78	1.47	0.66	0.77	2.00	2.46	0.00	0.81	2.34	0.86	1.51	0.63
AU526:B5	25	0.93	0.00	0.69	0.00	1.67	0.00	0.77	0.42	0.00	1.80	2.20	0.87
AU526:B6	20	2.32	1.91	0.00	0.00	2.08	3.20	0.00	0.53	3.05	0.56	1.96	0.81
Median	23	1.82	0.73	0.67	0.00	1.81	1.23	0.30	0.72	1.17	1.33	1.54	0.73
Mean	20	1.63	0.82	0.72	0.13	1.76	1.37	0.59	0.81	1.30	1.35	1.35	0.76
AU526:O1	0												
AU526:O2	37	0.00	1.03	2.78	1.62	0.85	0.00	0.78	1.28	0.00	0.61	0.42	1.17
AU526:O3	42	0.55	1.82	1.64	0.95	1.49	0.00	0.92	1.13	0.00	0.80	0.37	1.16
AU526:O4	49	0.47	2.34	2.45	1.22	1.28	0.00	0.79	0.97	0.00	0.69	0.32	1.22
AU526:O5	49	0.47	2.34	2.45	1.22	1.28	0.00	0.79	0.97	0.00	0.69	0.32	1.22
AU526:O6	51	0.45	2.25	2.36	1.18	1.43	0.00	0.75	0.93	0.00	0.66	0.31	1.28
Median	46	0.47	2.25	2.45	1.22	1.28	0.00	0.79	0.97	0.00	0.69	0.32	1.22
Mean	38	0.39	1.96	2.34	1.24	1.26	0.00	0.80	1.05	0.00	0.69	0.35	1.21
**AU526:Offspring**													
AU526:B1A	34	0.00	2.25	1.52	2.35	1.23	0.00	1.13	1.08	0.00	1.65	0.23	0.64
AU526:B2A	26	1.78	1.47	0.00	0.00	2.41	2.46	0.00	0.61	2.34	0.86	1.81	0.83
AU526:B3A	23	2.02	1.66	0.00	0.87	1.81	2.78	0.00	0.69	2.65	0.98	1.71	0.71
AU526:B4A	28	1.66	1.36	0.00	0.71	3.35	2.28	0.00	0.56	2.18	0.80	1.40	0.58
AU526:B5A	25	1.86	1.53	0.00	1.60	1.67	2.56	0.00	0.63	2.44	1.35	1.57	0.65
AU526:B6A	27	1.72	1.42	0.00	0.00	1.93	2.37	0.00	0.98	2.26	0.83	1.75	0.80
Median	27	1.75	1.50	0.00	0.79	1.87	2.41	0.00	0.66	2.30	0.92	1.64	0.68
Mean	27	1.51	1.61	0.25	0.92	2.07	2.07	0.19	0.76	1.98	1.08	1.41	0.70
AU526:O1A	49	0.95	0.78	1.05	2.04	0.85	1.30	0.79	1.29	2.49	0.69	0.64	0.89
AU526:O2A	49	0.47	2.34	2.45	1.22	1.49	0.00	0.79	0.97	0.00	0.69	0.32	1.11
AU526:O2B	51	0.45	3.00	2.69	1.18	1.23	0.00	0.75	0.93	0.00	0.66	0.31	1.17
AU526:O4A	52	0.45	2.20	2.31	1.15	1.60	0.00	0.74	0.91	0.00	0.65	0.30	1.25
Median	50	0.46	2.27	2.38	1.20	1.36	0.00	0.77	0.95	0.00	0.67	0.31	1.14
Mean	50	0.58	2.08	2.13	1.40	1.29	0.33	0.77	1.02	0.62	0.67	0.39	1.10

*Ratio values greater than 2.5 are shown on a dark gray background*.

Approximately 30% of substitutions were non-synonymous, resulting in a median of 6 amino acid differences (range, 4–7) between AU526 and isolates from pregnant heifers and a median of 13 amino acid differences (range, 0–14) between AU526 and isolates from pregnant ewes (**Table 6**). Similar to their dams, a median of 7 substitutions (range, 6–13) was detected in isolates from calves and a median of 15 substitutions (range, 13–17) was observed in isolates from lambs. The difference between the median number of amino acid changes observed in bovine and ovine isolates was statistically significant (*P* = 0.003). In bovine isolates, these substitutions occurred most frequently in the E2 and N^pro^ coding regions with 36% and 23% of changes in these regions, respectively (**Table 6**). In ovine isolates, 37, 19, and 16% of substitutions were found in the E2, NS5B, and E^rns^ coding regions, respectively. In all but two bovine isolates, the number of amino acid changes observed in the E2 and N^pro^ coding regions was at least 2.5 times greater than expected from random substitutions across the viral genome (**Table 7**). In all but four ovine isolates, amino acid changes occurred in the E^rns^ and E2 coding regions at least 2.5 times more frequently than expected from random events. In contrast, there were no amino acid substitutions in the NS4A coding region. Furthermore, there were only four isolates (O2, B6A, O1A, and O2B) with an amino acid change in regions encoding the viral proteins C, p7, and NS5A.

**Table 6:** Amino acid substitutions between AU526 and isolates from acutely infected dams and their offspring.

	Total	N^pro^	C	E^rns^	E1	E2	p7	NS2	NS3	NS4A	NS4B	NS5A	NS5B	% S*^a^*	% NS*^b^*
**AU526:Dam**															
AU526:B1	4	0	0	0	0	1	0	2	0	0	1	0	0	25.0	75.0
AU526:B2	7	1	0	1	0	3	0	0	0	0	1	0	1	57.1	42.9
AU526:B3	6	2	0	0	0	2	0	0	1	0	1	0	0	33.3	66.7
AU526:B4	6	2	0	0	1	2	0	0	1	0	0	0	0	50.0	50.0
AU526:B5	7	1	0	1	0	2	0	1	1	0	0	0	1	42.9	57.1
AU526:B6	5	2	0	0	0	2	0	0	1	0	0	0	0	40.0	60.0
Median	6	2	0	0	0	2	0	0	1	0	1	0	0	41.4	58.6
Mean	6	1	0	0	0	2	0	1	1	0	1	0	0	41.4	58.6
AU526:O1	0	0	0	0	0	0	0	0	0	0	0	0	0		
AU526:O2	13	0	0	3	2	2	0	2	0	0	0	1	3	53.8	46.2
AU526:O3	12	0	0	2	1	5	0	1	1	0	0	0	2	66.7	33.3
AU526:O4	13	0	0	2	1	5	0	1	1	0	0	0	3	61.5	38.5
AU526:O5	13	0	0	2	1	5	0	1	1	0	0	0	3	61.5	38.5
AU526:O6	14	0	0	2	1	6	0	1	1	0	0	0	3	64.3	35.7
Median	13	0	0	2	1	5	0	1	1	0	0	0	3	61.5	38.5
Mean	11	0	0	2	1	4	0	1	1	0	0	0	2	61.6	38.4
**AU526:Offspring**															
AU526:B1A	13	0	0	2	3	4	0	1	0	0	2	0	1	69.2	30.8
AU526:B2A	7	2	0	0	0	3	0	0	1	0	0	0	1	42.9	57.1
AU526:B3A	6	2	0	0	1	2	0	0	1	0	0	0	0	50.0	50.0
AU526:B4A	10	2	0	0	1	6	0	0	1	0	0	0	0	70.0	30.0
AU526:B5A	6	2	0	0	1	2	0	0	1	0	0	0	0	50.0	50.0
AU526:B6A	7	2	0	0	0	2	0	0	2	0	0	1	0	28.6	71.4
Median	7	2	0	0	1	3	0	0	1	0	0	0	0	50.0	50.0
Mean	8	2	0	0	1	3	0	0	1	0	0	0	0	51.8	48.2
AU526:O1A	17	1	0	2	3	4	1	3	0	0	1	0	2	52.9	47.1
AU526:O2A	13	0	0	2	1	6	0	1	1	0	0	0	2	69.2	30.8
AU526:O2B	14	0	1	3	1	5	0	1	1	0	0	0	2	71.4	28.6
AU526:O4A	15	0	0	2	1	7	0	1	1	0	0	0	3	66.7	33.3
Median	15	0	0	2	1	6	0	1	1	0	0	0	2	67.9	32.1
Mean	15	0	0	2	2	6	0	2	1	0	0	0	2	65.1	34.9

^a^*Percentage of substitutions within structural protein-coding regions. *^*b*^*Percentage of substitutions within non-structural protein-coding regions.*

**Table 7:** Ratio of the number of observed amino acid substitutions per protein-coding region to the number of expected substitutions assuming a random distribution across the viral genome.

	Total	N^pro^	C	E^rns^	E1	E2	p7	NS2	NS3	NS4A	NS4B	NS5A	NS5B
**AU526:Dam**													
AU526:B1	4	0.00	0.00	0.00	0.00	2.61	0.00	4.81	0.00	0.00	2.81	0.00	0.00
AU526:B2	7	3.31	0.00	2.45	0.00	4.47	0.00	0.00	0.00	0.00	1.60	0.00	0.77
AU526:B3	6	7.73	0.00	0.00	0.00	3.47	0.00	0.00	0.88	0.00	1.87	0.00	0.00
AU526:B4	6	7.73	0.00	0.00	3.33	3.47	0.00	0.00	0.88	0.00	0.00	0.00	0.00
AU526:B5	7	3.31	0.00	2.45	0.00	2.98	0.00	1.37	0.75	0.00	0.00	0.00	0.77
AU526:B6	5	9.28	0.00	0.00	0.00	4.17	0.00	0.00	1.05	0.00	0.00	0.00	0.00
Median	6	5.52	0.00	0.00	0.00	3.47	0.00	0.00	0.82	0.00	0.80	0.00	0.00
Mean	6	5.23	0.00	0.82	0.56	3.53	0.00	1.03	0.59	0.00	1.05	0.00	0.26
AU526:O1	0												
AU526:O2	13	0.00	0.00	3.96	3.08	1.60	0.00	1.48	0.00	0.00	0.00	0.60	1.25
AU526:O3	12	0.00	0.00	2.86	1.67	4.34	0.00	0.80	0.44	0.00	0.00	0.00	0.90
AU526:O4	13	0.00	0.00	2.64	1.54	4.01	0.00	0.74	0.41	0.00	0.00	0.00	1.25
AU526:O5	13	0.00	0.00	2.64	1.54	4.01	0.00	0.74	0.41	0.00	0.00	0.00	1.25
AU526:O6	14	0.00	0.00	2.45	1.43	4.47	0.00	0.69	0.38	0.00	0.00	0.00	1.16
Median	13	0.00	0.00	2.64	1.54	4.01	0.00	0.74	0.41	0.00	0.00	0.00	1.25
Mean	11	0.00	0.00	2.91	1.85	3.69	0.00	0.89	0.33	0.00	0.00	0.12	1.16
**AU526:Offspring**													
AU526:B1A	13	0.00	0.00	2.64	4.61	3.21	0.00	0.74	0.00	0.00	1.73	0.00	0.42
AU526:B2A	7	6.63	0.00	0.00	0.00	4.47	0.00	0.00	0.75	0.00	0.00	0.00	0.77
AU526:B3A	6	7.73	0.00	0.00	3.33	3.47	0.00	0.00	0.88	0.00	0.00	0.00	0.00
AU526:B4A	10	4.64	0.00	0.00	2.00	6.25	0.00	0.00	0.53	0.00	0.00	0.00	0.00
AU526:B5A	6	7.73	0.00	0.00	3.33	3.47	0.00	0.00	0.88	0.00	0.00	0.00	0.00
AU526:B6A	7	6.63	0.00	0.00	0.00	2.98	0.00	0.00	1.51	0.00	0.00	1.12	0.00
Median	7	6.63	0.00	0.00	2.67	3.47	0.00	0.00	0.82	0.00	0.00	0.00	0.00
Mean	8	5.56	0.00	0.44	2.21	3.98	0.00	0.12	0.76	0.00	0.29	0.19	0.20
AU526:O1A	17	1.36	0.00	2.02	3.53	2.45	3.76	1.70	0.00	0.00	0.66	0.00	0.64
AU526:O2A	13	0.00	0.00	2.64	1.54	4.81	0.00	0.74	0.41	0.00	0.00	0.00	0.83
AU526:O2B	14	0.00	2.73	3.68	1.43	3.72	0.00	0.69	0.38	0.00	0.00	0.00	0.77
AU526:O4A	15	0.00	0.00	2.29	1.33	4.86	0.00	0.64	0.35	0.00	0.00	0.00	1.08
Median	15	0.00	0.00	2.47	1.48	4.27	0.00	0.71	0.36	0.00	0.00	0.00	0.80
Mean	15	0.34	0.68	2.66	1.96	3.96	0.94	0.94	0.28	0.00	0.17	0.00	0.83

*Ratio values greater than 2.5 are shown on a dark gray background*.

**FIGURE 1:**
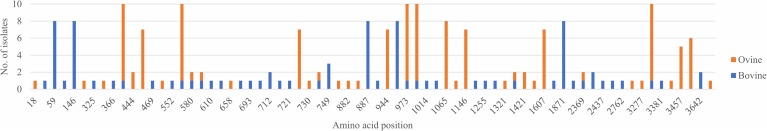
Location of all amino acid substitutions identified in bovine and ovine isolates. For each amino acid residue, the number of isolates that had a substitution at that position is given. Positions are numbered relative to the polyprotein of BVDV-1b AU526 (MG950344).

A total of 284 nucleotide substitutions occurred in bovine isolates at 125 different positions, whereas 429 substitutions occurred in ovine isolates at 109 different positions. There were also 84 amino acid substitutions at 44 different positions in bovine isolates and 124 substitutions at 36 different positions in ovine isolates (**Figure 1**). Amino acid residues that were often affected by substitutions in ovine isolates were rarely substituted in bovine isolates and vice versa. Furthermore, the number of nucleotide and amino positions affected by substitutions in bovine isolates was statistically different from that observed in ovine isolates (*P* < 0.001 and *P* = 0.025, respectively). However, there was no statistical evidence that the number of nucleotide and amino acid positions affected by substitutions was different between isolates from pregnant dams and those from their offspring (*P* = 0.59 and *P* = 0.87, respectively).

Multiple nucleotide and amino acid changes introduced during the first and second passages in pregnant dams were found to be conserved in the remaining passages. In cattle, five amino acid substitutions detected in the isolate from the third heifer (B3) were also observed in the isolates from the fourth and sixth heifers and the last four calves (**Table 8**). Two of these changes occurred in both the N^pro^ and E2 coding regions. In sheep, five amino acid changes detected in the isolate from the second ewe (O2) were also observed in the isolates from the last four ewes and their lambs (**Table 9**). Seven additional amino acid changes identified in the isolate from the third ewe (O3) were also detected in the isolates from the subsequent ewes and their offspring. One additional substitution was shared by the isolates from the lambs of the second ewe (O2A and O2B) and the isolates from the last three ewes. In summary, a total of 13 amino acid substitutions were conserved in the isolates from the last three ewes and their lambs. These changes occurred most frequently in regions encoding the viral proteins E2, NS5B, and E^rns^ with four, three, and two changes in these regions, respectively.

**Table 8:** Conserved amino acid substitutions during serial infection of pregnant heifers with BVDV in early pregnancy.

Viral protein	N^pro^		E2		NS3
AA position	59	146	887	946	1871
AU526	D	K	L	R	D
B1	D	K	L	R	D
B1A	D	K	L	R	D
B2	D	K	L	R	D
B2A	N	R	A	R	E
B3	N	R	V	Q	E
B3A	N	R	V	Q	E
B4	N	R	V	Q	E
B4A	N	R	V	Q	E
B5	D	K	L	Q	D
B5A	N	R	V	Q	E
B6	N	R	V	Q	E
B6A	N	R	V	Q	E

*Conserved amino acid (AA) substitutions are shown on a dark gray background*.

**Table 9:** Conserved amino acid substitutions during serial infection of pregnant ewes with BVDV in early pregnancy.

Viral protein	E^rns^		E1	E2				p7	NS2	NS3	NS5b		
AA position	439	453	554	728	944	973	993	1065	1146	1607	3284	3457	3611
AU526	H	V	V	G	T	Y	R	S	R	M	R	I	I
O1	H	V	V	G	T	Y	R	S	R	M	R	I	I
O1A	D	V	M	G	T	H	K	L	R	M	K	I	I
O2	D	V	M	G	T	H	K	S	R	M	K	I	I
O2A	D	I	M	R	N	H	K	L	Q	I	K	I	V
O2B	D	I	M	R	N	H	K	L	Q	I	K	I	V
O3	D	I	M	R	N	H	K	L	Q	I	K	V	I
O4	D	I	M	R	N	H	K	L	Q	I	K	V	V
O4A	D	I	M	R	N	H	K	L	Q	I	K	V	V
O5	D	I	M	R	N	H	K	L	Q	I	K	V	V
O6	D	I	M	R	N	H	K	L	Q	I	K	V	V

*Conserved amino acid (AA) substitutions are shown on a dark gray background*.

On average 43% of nucleotide and 42% of amino acid differences between AU526 and isolates from calves were observed in isolates from their dams (**Table 10**). Similarly, on average 39% of nucleotide and 40% of amino acid differences between AU526 and isolates from lambs were also observed in isolates from their dams. Of these amino acid differences, an average of 54 and 74% occurred in structural protein-coding regions in isolates from calves and lambs, respectively. In comparison, on average 56 and 63% of changes in isolates from heifers and ewes that were not detected in their offspring occurred in regions encoding non-structural proteins, respectively (**Table 11**).

**Table 10:** Substitutions between AU526 and isolates from offspring that were detected in isolates from their acutely infected dams.

AU526:Offspring	No of nucleotide substitutions	%	%S*^a^*	%NS*^b^*	No of amino acid substitutions	%	%S*^a^*	%NS*^b^*
AU526:B1A	0	0.0			0	0.0		
AU526:B2A	2	7.7	50.0	50.0	2	28.6	50.0	50.0
AU526:B3A	22	95.7	22.7	77.3	5	83.3	40.0	60.0
AU526:B4A	22	78.6	22.7	77.3	5	50.0	40.0	60.0
AU526:B5A	1	4.0	100.0	0.0	1	16.7	100.0	0.0
AU526:B6A	20	74.1	25.0	75.0	5	71.4	40.0	60.0
Median	11	40.9	25.0	75.0	4	39.3	40.0	60.0
Mean	11	43.3	44.1	55.9	3	41.7	54.0	46.0
AU526:O1A	0	0.0			0	0.0		
AU526:O2A	15	30.6	46.7	53.3	5	38.5	80.0	20.0
AU526:O2B	15	29.4	46.7	53.3	5	35.7	80.0	20.0
AU526:O4A	49	94.2	38.8	61.2	13	86.7	61.5	38.5
Median	15	30.0	46.7	53.3	5	37.1	80.0	20.0
Mean	20	38.6	44.0	56.0	6	40.2	73.8	26.2

*^*a*^*Percentage of substitutions within structural protein-coding regions. *^*b*^*Percentage of substitutions within non-structural protein-coding regions.

**Table 11:** Substitutions between AU526 and isolates from acutely infected dams that were not detected in isolates from their offspring.

AU526:Dam	No of nucleotide substitutions	%	%S*^a^*	%NS*^b^*	No of amino acid substitutions	%	%S*^a^*	%NS*^b^*
AU526:B1	9	100.0	22.2	77.8	4	100.0	25.0	75.0
AU526:B2	14	87.5	21.4	78.6	5	71.4	60.0	40.0
AU526:B3	3	12.0	0.0	100.0	1	16.7	0.0	100.0
AU526:B4	4	15.4	75.0	25.0	1	16.7	100.0	0.0
AU526:B5	24	96.0	16.7	83.3	6	85.7	33.3	66.7
AU526:B6	0	0.0			0	0.0		
Median	7	51.4	21.4	78.6	3	44.0	33.3	66.7
Mean	9	51.8	27.1	72.9	3	48.4	43.7	56.3
AU526:O1	0				0			
AU526:O2*^c^*	22	59.5	27.3	72.7	8	61.5	37.5	62.5
AU526:O4	0	0.0			0	0.0		
Median	0	29.8	27.3	72.7	0	30.8	37.5	62.5
Mean	7	29.8	27.3	72.7	3	30.8	37.5	62.5

^a^*Percentage of substitutions within structural protein-coding regions. *^*b*^*Percentage of substitutions within non-structural protein-coding regions. *^*c*^*Identical results were obtained for the two lambs of the second ewe (O2A and O2B)*.

To investigate temporal changes in viral populations circulating in PI calves, VI-positive serum samples obtained at 42, 84, and 168 days of age from live PI calves were also used in genomic sequencing procedures. The complete ORF sequences of eight BVDV-1b isolates were thus determined. Two sequences contained one ambiguous nucleotide. There was a median of 3 nucleotide and 3 amino acid substitutions between viruses isolated from the first VI-positive serum samples collected after birth and those from serum samples collected at later time points (**Table 12**). Furthermore, all but one amino acid substitutions occurred in regions encoding the viral structural proteins E1 and E2.

**Table 12:** Substitutions between isolates obtained from persistently infected calves at different time points during the first 6 months of life.

Offspring	No of nucleotide substitutions	%S*^a^*	%NS*^b^*	No of amino acid substitutions	%S*^a^*	%NS*^b^*
B2A (84:168 doa)	4	50.0	50.0	3	66.7	33.3
B3A (birth:168 doa)	3	100.0	0.0	3	100.0	0.0
B4A (42:84 doa)	6	100.0	0.0	5	100.0	0.0
B4A (42:168 doa)	7	100.0	0.0	6	100.0	0.0
B5A (42:84 doa)	0			0		
B5A (42:168 doa)	2	100.0	0.0	2	100.0	0.0
B6A (42:84 doa)	0			0		
B6A (42:168 doa)	0			0		
Median	3	100.0	0.0	3	100.0	0.0
Mean	3	90.0	10.0	2	93.3	6.7

^a^*Percentage of substitutions within structural protein-coding regions. *^*b*^*Percentage of substitutions within non-structural protein-coding regions. doa, days of age*.

Results of all pairwise sequence comparisons as well as the location and type of nucleotide and amino acid substitutions between AU526 and isolates from acutely infected pregnant dams and their offspring are given in the Supplementary Tables S1–S6. Furthermore, virus titers in samples from pregnant dams and their offspring as well as the results of qRT-PCR analyses of serum samples from pregnant ewes are given in the Supplementary Tables S7–S9.

## Discussion

In this study, the BVDV-1b isolate AU526 was serially passaged in six pregnant heifers and six pregnant ewes. Acute maternal infections resulted in transplacental transmission of BVDV and the birth of six calves and four lambs, from which virus could be isolated. The complete ORF sequences of AU526 and isolates from pregnant dams and their offspring were obtained and compared to determine the timing, number, location, and type of substitutions introduced during serial infection of pregnant cattle and sheep with BVDV.

The median number of nucleotide and amino acid differences between AU526 and isolates from pregnant heifers was similar to the median of 20 nucleotide and 8 amino acid changes observed in isolates from four pregnant cows exposed to calves PI with BVDV (Neill et al., 2012). Compared to cattle, greater numbers of changes were detected between AU526 and isolates from pregnant ewes with a median of 46 nucleotide and 13 amino acid substitutions. Similarly, 10 and 36 nucleotide substitutions were detected in isolates from two pregnant does acutely infected with BVDV (Passler et al., 2014). When comparing AU526 to isolates from offspring born to these dams, similar findings were observed with more changes in isolates from lambs than in those obtained from calves. Altogether, these results revealed that many substitutions were introduced in the BVDV genome during the establishment of serial persistent infections in sheep and suggest that BVDV infections in pregnant small ruminants may serve as a significant source of viral genetic diversity. Previous studies demonstrated severe reduction in RNA virus populations during vector-borne transmission and when viruses spread within a single host (Navas et al., 1998; Forrester et al., 2012; Dow et al., 2015). There are several host barriers and defense mechanisms that limit virus infection and spread in a heterologous host and therefore cross-species infections are likely to impose severe bottlenecks on virus populations. It is thus possible that serial infection of pregnant sheep with a BVDV isolate of bovine origin was associated with greater numbers of substitutions due to high selection pressure on virus populations.

Although greater numbers of changes occurred during serial infection of pregnant sheep, the number of different nucleotide and amino acid positions affected by substitutions was greater in bovine isolates than in ovine isolates. It is possible that substitutions were more easily tolerated in the host of origin or that more diverse changes were introduced in cattle because host factors affected the activity of the BVDV polymerase complex. Furthermore, acute BVDV infections in pregnant cattle may result in the birth of PI calves. Considering that PI calves consistently shed large amounts of virus and that additional substitutions were detected in isolates from calves, these animals may serve as an important source of viral isolates that are genetically diverse from those infecting their dams. Altogether these results suggest that acute infections in pregnant cattle may greatly contribute to the generation and spread of new BVDV isolates in animal populations.

Nucleotide substitutions occurred randomly throughout the BVDV genome in bovine and ovine isolates. However, there was a bias toward amino acid changes in the E2 and N^pro^ coding regions in bovine isolates and the E2 and E^rns^ in ovine isolates. Previous studies have also demonstrated that amino acid changes occurred primarily in regions encoding the structural proteins E2 and E^rns^ during the establishment of persistent infections in cattle (Neill et al., 2011, 2012). The envelope proteins E2 and E^rns^ are the main targets of neutralizing antibodies in animals infected with BVDV and immune pressure on the immunodominant residues may have contributed to the selection of amino acid changes in these coding regions. Furthermore, amino acid changes in the N^pro^ coding region were anticipated since this region of the viral genome is relatively variable across pestivirus genomes and within PI cattle (Smith et al., 2017; Chernick et al., 2018).

Detailed examination of the genomic sequences revealed that multiple amino acid substitutions were gradually introduced and conserved during serial infection of pregnant cattle and sheep with BVDV. A total of five amino acid changes were shared by all but one of the isolates from the last four heifers and their calves. Analogously, 12 to 13 identical changes were detected in the isolates from the last four ewes and their lambs. The amino acid residues involved were different from one species to another and these changes were most frequently observed in the E2 coding region. These conserved substitutions may thus be involved in host adaptation. Host-specific amino acid changes in the E2 coding region were described during serial infection of pregnant cattle and sheep (Paton et al., 1997). Conserved amino acid changes in the E2 and E^rns^ coding regions were also reported in BVDV-1b isolates from alpacas (Neill et al., 2015). Analogously, most conserved changes detected in ovine isolates in this study occurred in the same coding regions of the BVDV genome.

An unexpected finding was the presence of some of these conserved changes in isolates from offspring (B2A, B5A, O1A, O2A, and O2B) that were born to dams whose isolates did not have these changes. This finding suggests that similar selection mechanisms may be present in acutely infected pregnant animals and PI offspring. It is currently unknown, and will be the focus of future experiments, if these host-specific changes provided a selective advantage in infected sheep and if similar results would have been obtained if a BVDV isolate belonging to a different subgenotype or species had been serially passaged in sheep.

In contrast to a study conducted in cattle (Neill et al., 2012), most nucleotide and amino acid changes identified in isolates from calves and lambs were not detected in isolates from their acutely infected dams. However, there was a high degree of variation between offspring. For instance, 96% of nucleotide substitutions identified in the isolate of the calf B3A were detected in the isolate of its dam. Similar findings were reported in goats with 95% of nucleotide substitutions observed in the isolate of a PI goat kid that were detected in the isolate of its dam whereas 60% of changes observed in the isolate of another PI goat kid were not detected in the isolate of its dam (Passler et al., 2014). Considering the existence of a viral quasispecies in PI cattle, it is possible that the substitutions observed in isolates from offspring might have been first established in pregnant dams in a limited number of viral mutants that were subsequently selected in PI offspring (Dow et al., 2015; Ridpath et al., 2015). Fluctuations in minor variants within the viral quasispecies may partially explain the discrepancies observed between and within studies performed in cattle and other species. This hypothesis could not be confirmed or excluded since our analysis was restricted to the consensus sequence of the BVDV ORF.

The majority of the amino acid substitutions present in isolates from acutely infected dams that were also detected in isolates from their offspring occurred in regions encoding structural viral proteins. In contrast, amino acid changes in isolates from pregnant dams that were not observed in isolates from their offspring occurred primarily in non-structural protein-coding regions. These results suggest that amino acid substitutions in regions encoding structural viral proteins were positively selected during establishment of persistent infection in offspring. It is possible that these changes were associated with fitness gain in PI offspring.

It is possible that viral populations circulating in PI animals evolve during the first months of life, similar to what has been described in children congenitally infected with hepatitis C virus (Fauteux-Daniel et al., 2017). In this study, small numbers of nucleotide and amino acid differences were observed between viruses that were isolated from PI calves at different time points during the first 6 months of life. Similar results have been reported in 34 PI cattle that were sampled at three different time points during the first year of life (Ridpath et al., 2015).

## Conclusion

In conclusion, greater numbers of nucleotide and amino acid substitutions were introduced during serial infection of pregnant sheep than of pregnant cattle. Furthermore, multiple host-specific amino acid changes were gradually introduced and conserved in bovine and ovine isolates. These changes were primarily in the E2 coding region and more abundant in ovine isolates. These results suggest that BVDV infections in heterologous hosts may serve as a significant source of viral genetic diversity and may be associated with adaptive changes.

## Author Contributions

All authors listed have made a substantial, direct and intellectual contribution to the work, and approved it for publication.

## Conflict of Interest Statement

The authors declare that the research was conducted in the absence of any commercial or financial relationships that could be construed as a potential conflict of interest.

## Footnotes

1 http://www.megasoftware.net
