# Supplementary material for: Identification of Conserved Amino Acid Substitutions During Serial Infection of Pregnant Cattle and Sheep With Bovine Viral Diarrhea Virus
Source: Front Microbiol. 2018 Jun 6;9:1109. doi: 10.3389/fmicb.2018.01109 (PMC5998738; doi:10.3389/fmicb.2018.01109)
Supplement: Supplementary file 1 [file Table_1.docx]

Supplementary Material

**Identification of Conserved Amino Acid Substitutions**

**during Serial Infection of Pregnant Cattle and Sheep**

**with Bovine Viral Diarrhea Virus**

Thibaud Kuca^1^, Thomas Passler^1*^, Benjamin W. Newcomer^1^, John D. Neill^2^, Patricia K. Galik^3^, Kay P. Riddell^3^, Yijing Zhang^3^ and Paul H. Walz^3^

*** Correspondence:** Thomas Passler: passlth@auburn.edu

**Table S1.** Number of nucleotide differences between AU526 and isolates from acutely infected dams and their offspring.

|  | AU526 | B1 | B1A | B2 | B2A | B3 | B3A | B4 | B4A | B5 | B5A | B6 | B6A | O1 | O1A | O2 | O2A | O2B | O3 | O4 | O4A | O5 |
| --- | --- | --- | --- | --- | --- | --- | --- | --- | --- | --- | --- | --- | --- | --- | --- | --- | --- | --- | --- | --- | --- | --- |
| B1 | 9 |  |  |  |  |  |  |  |  |  |  |  |  |  |  |  |  |  |  |  |  |  |
| B1A | 34 | 41 |  |  |  |  |  |  |  |  |  |  |  |  |  |  |  |  |  |  |  |  |
| B2 | 16 | 21 | 48 |  |  |  |  |  |  |  |  |  |  |  |  |  |  |  |  |  |  |  |
| B2A | 26 | 31 | 60 | 38 |  |  |  |  |  |  |  |  |  |  |  |  |  |  |  |  |  |  |
| B3 | 25 | 32 | 59 | 41 | 9 |  |  |  |  |  |  |  |  |  |  |  |  |  |  |  |  |  |
| B3A | 23 | 30 | 57 | 39 | 7 | 4 |  |  |  |  |  |  |  |  |  |  |  |  |  |  |  |  |
| B4 | 26 | 33 | 60 | 42 | 10 | 7 | 5 |  |  |  |  |  |  |  |  |  |  |  |  |  |  |  |
| B4A | 28 | 35 | 60 | 44 | 12 | 9 | 7 | 10 |  |  |  |  |  |  |  |  |  |  |  |  |  |  |
| B5 | 25 | 34 | 59 | 41 | 51 | 48 | 46 | 49 | 51 |  |  |  |  |  |  |  |  |  |  |  |  |  |
| B5A | 25 | 32 | 59 | 41 | 9 | 6 | 4 | 7 | 9 | 48 |  |  |  |  |  |  |  |  |  |  |  |  |
| B6 | 20 | 27 | 54 | 36 | 8 | 5 | 3 | 6 | 8 | 43 | 5 |  |  |  |  |  |  |  |  |  |  |  |
| B6A | 27 | 34 | 61 | 43 | 11 | 8 | 6 | 9 | 11 | 50 | 8 | 7 |  |  |  |  |  |  |  |  |  |  |
| O1 | 0 | 9 | 33 | 16 | 26 | 25 | 23 | 26 | 28 | 25 | 25 | 20 | 27 |  |  |  |  |  |  |  |  |  |
| O1A | 49 | 58 | 47 | 63 | 75 | 74 | 72 | 73 | 77 | 74 | 74 | 69 | 74 | 48 |  |  |  |  |  |  |  |  |
| O2 | 37 | 46 | 43 | 51 | 63 | 62 | 60 | 63 | 65 | 62 | 62 | 57 | 64 | 37 | 50 |  |  |  |  |  |  |  |
| O2A | 49 | 58 | 57 | 63 | 73 | 72 | 70 | 73 | 75 | 74 | 72 | 67 | 74 | 49 | 64 | 56 |  |  |  |  |  |  |
| O2B | 51 | 60 | 59 | 65 | 75 | 74 | 72 | 75 | 77 | 76 | 74 | 69 | 76 | 51 | 66 | 58 | 4 |  |  |  |  |  |
| O3 | 42 | 51 | 52 | 56 | 66 | 65 | 63 | 66 | 68 | 67 | 65 | 60 | 67 | 42 | 59 | 51 | 7 | 9 |  |  |  |  |
| O4 | 49 | 58 | 57 | 63 | 73 | 72 | 70 | 73 | 75 | 74 | 72 | 67 | 74 | 49 | 64 | 56 | 2 | 4 | 5 |  |  |  |
| O4A | 52 | 61 | 60 | 66 | 76 | 75 | 73 | 76 | 78 | 77 | 75 | 70 | 77 | 52 | 67 | 59 | 5 | 7 | 8 | 3 |  |  |
| O5 | 49 | 58 | 57 | 63 | 73 | 72 | 70 | 73 | 75 | 74 | 72 | 67 | 74 | 49 | 64 | 56 | 2 | 4 | 5 | 0 | 3 |  |
| O6 | 51 | 60 | 59 | 65 | 75 | 74 | 72 | 75 | 77 | 76 | 74 | 69 | 76 | 51 | 66 | 58 | 4 | 6 | 7 | 2 | 5 | 2 |

**Table S2.** Number of amino acid differences between AU526 and isolates from acutely infected dams and their offspring.

|  | AU526 | B1 | B1A | B2 | B2A | B3 | B3A | B4 | B4A | B5 | B5A | B6 | B6A | O1 | O1A | O2 | O2A | O2B | O3 | O4 | O4A | O5 |
| --- | --- | --- | --- | --- | --- | --- | --- | --- | --- | --- | --- | --- | --- | --- | --- | --- | --- | --- | --- | --- | --- | --- |
| B1 | 4 |  |  |  |  |  |  |  |  |  |  |  |  |  |  |  |  |  |  |  |  |  |
| B1A | 13 | 17 |  |  |  |  |  |  |  |  |  |  |  |  |  |  |  |  |  |  |  |  |
| B2 | 7 | 7 | 20 |  |  |  |  |  |  |  |  |  |  |  |  |  |  |  |  |  |  |  |
| B2A | 7 | 9 | 20 | 10 |  |  |  |  |  |  |  |  |  |  |  |  |  |  |  |  |  |  |
| B3 | 6 | 10 | 19 | 13 | 6 |  |  |  |  |  |  |  |  |  |  |  |  |  |  |  |  |  |
| B3A | 6 | 10 | 19 | 13 | 6 | 2 |  |  |  |  |  |  |  |  |  |  |  |  |  |  |  |  |
| B4 | 6 | 10 | 19 | 13 | 6 | 2 | 2 |  |  |  |  |  |  |  |  |  |  |  |  |  |  |  |
| B4A | 10 | 14 | 21 | 17 | 10 | 6 | 6 | 6 |  |  |  |  |  |  |  |  |  |  |  |  |  |  |
| B5 | 7 | 11 | 20 | 14 | 14 | 11 | 11 | 11 | 15 |  |  |  |  |  |  |  |  |  |  |  |  |  |
| B5A | 6 | 10 | 19 | 13 | 6 | 2 | 2 | 2 | 6 | 11 |  |  |  |  |  |  |  |  |  |  |  |  |
| B6 | 5 | 9 | 18 | 12 | 5 | 1 | 1 | 1 | 5 | 10 | 1 |  |  |  |  |  |  |  |  |  |  |  |
| B6A | 7 | 11 | 20 | 14 | 7 | 3 | 3 | 3 | 7 | 12 | 3 | 2 |  |  |  |  |  |  |  |  |  |  |
| O1 | 0 | 4 | 12 | 7 | 7 | 6 | 6 | 6 | 10 | 7 | 6 | 5 | 7 |  |  |  |  |  |  |  |  |  |
| O1A | 17 | 21 | 12 | 24 | 24 | 23 | 23 | 21 | 27 | 24 | 23 | 22 | 24 | 16 |  |  |  |  |  |  |  |  |
| O2 | 13 | 17 | 16 | 20 | 20 | 19 | 19 | 19 | 23 | 20 | 19 | 18 | 20 | 13 | 16 |  |  |  |  |  |  |  |
| O2A | 13 | 17 | 16 | 20 | 20 | 19 | 19 | 19 | 23 | 20 | 19 | 18 | 20 | 13 | 18 | 16 |  |  |  |  |  |  |
| O2B | 14 | 18 | 17 | 21 | 21 | 20 | 20 | 20 | 24 | 21 | 20 | 19 | 21 | 14 | 19 | 17 | 3 |  |  |  |  |  |
| O3 | 12 | 16 | 15 | 19 | 19 | 18 | 18 | 18 | 22 | 19 | 18 | 17 | 19 | 12 | 17 | 15 | 3 | 4 |  |  |  |  |
| O4 | 13 | 17 | 16 | 20 | 20 | 19 | 19 | 19 | 23 | 20 | 19 | 18 | 20 | 13 | 18 | 16 | 2 | 3 | 1 |  |  |  |
| O4A | 15 | 19 | 18 | 22 | 22 | 21 | 21 | 21 | 25 | 22 | 21 | 20 | 22 | 15 | 20 | 18 | 4 | 5 | 3 | 2 |  |  |
| O5 | 13 | 17 | 16 | 20 | 20 | 19 | 19 | 19 | 23 | 20 | 19 | 18 | 20 | 13 | 18 | 16 | 2 | 3 | 1 | 0 | 2 |  |
| O6 | 14 | 18 | 17 | 21 | 21 | 20 | 20 | 20 | 24 | 21 | 20 | 19 | 21 | 14 | 19 | 17 | 3 | 4 | 2 | 1 | 3 | 1 |

**Table S3.** Location and type of nucleotide and amino acid substitutions between AU526 and isolates from acutely infected dams.

| AU526:Dam | Total | Location and type of nucleotide substitutions | Total | Location and type of amino acid substitutions |
| --- | --- | --- | --- | --- |
| AU526:B1 | 9 | 1017 C:T, **2246 C:T**, **3763 T:C**, **3841 G:C**, 5649 C:T, 5904 T:C, **7120 G:A**, 9138 T:C, 11328 G:A | 4 | 749 T:I, 1255 F:L, 1281 A:P, 2374 E:K |
| AU526:B2 | 16 | 133 C:T, **287 C:A**, **1096 A:G**, **2078 T:C**, **2146 A:G**, **2246 C:T**, 4467 A:G, 5223 C:A, 5733 A:G, 5982 G:A, 6534 C:A, **7120 G:A**, 7788 T:C, 9552 C:A, 10863 G:A, **10924 G:T** | 7 | 96 P:H, 366 K:E, 693 F:S, 716 T:A, 749 T:I, 2374 E:K, 3642 A:S |
| AU526:B3 | 25 | **175 G:A**, **437 A:G**, 549 G:A, 2208 T:C, 2439 A:G, **2659 C:G**, **2837 G:A**, 3292 C:T, **5613 C:A**, 5712 T:C, 6180 G:A, 6819 C:A, 7047 G:A, 7392 T:A, 7416 A:C, **7559 G:A**, 7746 C:T, 8182 C:T, 8769 A:G, 9024 C:T, 9138 T:C, 9327 T:C, 10044 C:T, 10068 A:G, 10479 G:A | 6 | 59 D:N, 146 K:R, 887 L:V, 946 R:Q, 1871 D:E, 2520 R:K |
| AU526:B4 | 26 | **175 G:A**, **437 A:G**, 549 G:A, 912 G:A, **1738 T:A**, 2208 T:C, 2439 A:G, 2562 C:T, **2659 C:G**, **2837 G:A**, 3292 C:T, **5613 C:A**, 5712 T:C, 6171 C:T, 6180 G:A, 6819 C:A, 7047 G:A, 7392 T:A, 8182 C:T, 8769 A:G, 9024 C:T, 9138 T:C, 9327 T:C, 10044 C:T, 10068 A:G, 10479 G:A | 6 | 59 D:N, 146 K:R, 580 S:T, 887 L:V, 946 R:Q, 1871 D:E |
| AU526:B5 | 25 | **83 C:T**, **975 C:A**, 2475 A:G, **2714 C:T**, 2739 C:T, **2837 G:A**, **3494 G:A**, 3789 A:G, **5179 A:G**, 6234 C:T, 7467 T:C, 7623 A:G, 7707 C:T, 8013 A:G, 8073 G:A, 8517 A:G, 8520 T:C, 8736 C:T, 9285 C:T, 9315 C:T, 9369 T:C, **10141 C:A**, 10455 G:A, 10683 T:C, 11577 G:A | 7 | 28 A:V, 325 H:Q, 905 S:F, 946 R:Q, 1165 R:K, 1727 T:A, 3381 H:N |
| AU526:B6 | 20 | **175 G:A**, **437 A:G**, 549 G:A, 2208 T:C, 2439 A:G, **2659 C:G**, **2837 G:A**, 3292 C:T, **5613 C:A**, 6180 G:A, 6819 C:A, 7047 G:A, 8182 C:T, 8769 A:G, 9024 C:T, 9138 T:C, 9327 T:C, 10044 C:T, 10068 A:G, 10479 G:A | 5 | 59 D:N, 146 K:R, 887 L:V, 946 R:Q, 1871 D:E |
| AU526:O1 | 0 |  | 0 |  |
| AU526:O2 | 37 | 783 C:T, **988 G:A**, 1008 T:C, 1203 T:C, 1245 C:A, **1315 C:G**, **1330 G:T**, 1626 A:G, **1660 G:A**, **1973 C:A**, **2917 T:C**, **2978 G:A**, 3174 C:T, 3720 C:T, **3962 A:G**, **4261 A:G**, 4701 A:G, 4881 G:T, 4956 C:T, 5148 G:A, 5181 G:A, 5562 A:G, 5733 A:G, 6009 A:G, 6372 A:G, 7524 T:C, 7836 A:G, 8451 A:C, **9034 G:A**, **9851 G:A**, 10005 T:C, 10245 G:T, **10248 G:T**, 10947 G:A, 11301 C:T, 11469 T:C, **11603 T:A** | 13 | 330 A:T, 439 H:D, 444 V:L, 554 V:M, 658 T:N, 973 Y:H, 993 R:K, 1321 K:R, 1421 T:A, 3012 A:T, 3284 R:K, 3416 E:D, 3868 V:E |
| AU526:O3 | 42 | 247 T:C, 669 A:G, 783 C:T, 846 A:G, 1254 A:G, **1315 C:G**, **1357 G:A**, 1626 A:G, **1660 G:A**, **2182 G:C**, 2451 C:A, **2831 C:A**, **2917 T:C**, **2978 G:A**, **3194 C:T**, **3437 G:A**, 3531 T:A, 3792 G:A, 3846 A:G, 4701 A:G, **4821 G:A**, 4881 G:T, 4959 A:T, 5016 T:C, 5148 G:A, 5562 A:G, 5679 A:G, 5733 A:G, 7524 T:C, 7725 T:A, 7962 A:G, 8461 T:C, 9408 T:C, **9851 G:A**, 10002 C:A, 10044 C:T, **10369 A:G**, 10407 T:C, 10911 A:G, 10947 G:A, 11049 T:C, 11094 T:C | 12 | 439 H:D, 453 V:I, 554 V:M, 728 G:R, 944 T:N, 973 Y:H, 993 R:K, 1065 S:L, 1146 R:Q, 1607 M:I, 3284 R:K, 3457 I:V |
| AU526:O4 | 49 | 247 T:C, 669 A:G, 726 A:G, 783 C:T, 846 A:G, 978 T:C, 1008 T:C, 1254 A:G, **1315 C:G**, **1357 G:A**, 1446 A:G, 1626 A:G, **1660 G:A**, 1812 A:G, **2182 G:C**, 2451 C:A, **2831 C:A**, **2917 T:C**, **2978 G:A**, **3194 C:T**, **3437 G:A**, 3531 T:A, 3792 G:A, 3846 A:G, 4701 A:G, **4821 G:A**, 4881 G:T, 4959 A:T, 5016 T:C, 5148 G:A, 5562 A:G, 5679 A:G, 5733 A:G, 7524 T:C, 7725 T:A, 7962 A:G, 8461 T:C, 9408 T:C, **9851 G:A**, 9927 T:C, 10002 C:A, 10044 C:T, **10369 A:G**, 10407 T:C, **10831 A:G**, 10911 A:G, 10947 G:A, 11049 T:C, 11094 T:C | 13 | 439 H:D, 453 V:I, 554 V:M, 728 G:R, 944 T:N, 973 Y:H, 993 R:K, 1065 S:L, 1146 R:Q, 1607 M:I, 3284 R:K, 3457 I:V, 3611 I:V |
| AU526:O5 | 49 | 247 T:C, 669 A:G, 726 A:G, 783 C:T, 846 A:G, 978 T:C, 1008 T:C, 1254 A:G, **1315 C:G**, **1357 G:A**, 1446 A:G, 1626 A:G, **1660 G:A**, 1812 A:G, **2182 G:C**, 2451 C:A, **2831 C:A**, **2917 T:C**, **2978 G:A**, **3194 C:T**, **3437 G:A**, 3531 T:A, 3792 G:A, 3846 A:G, 4701 A:G, **4821 G:A**, 4881 G:T, 4959 A:T, 5016 T:C, 5148 G:A, 5562 A:G, 5679 A:G, 5733 A:G, 7524 T:C, 7725 T:A, 7962 A:G, 8461 T:C, 9408 T:C, **9851 G:A**, 9927 T:C, 10002 C:A, 10044 C:T, **10369 A:G**, 10407 T:C, **10831 A:G**, 10911 A:G, 10947 G:A, 11049 T:C, 11094 T:C | 13 | 439 H:D, 453 V:I, 554 V:M, 728 G:R, 944 T:N, 973 Y:H, 993 R:K, 1065 S:L, 1146 R:Q, 1607 M:I, 3284 R:K, 3457 I:V, 3611 I:V |
| AU526:O6 | 51 | 247 T:C, 669 A:G, 726 A:G, 783 C:T, 846 A:G, 978 T:C, 1008 T:C, 1254 A:G, **1315 C:G**, **1357 G:A**, 1446 A:G, 1626 A:G, **1660 G:A**, 1812 A:G, **2182 G:C**, **2300 C:T**, 2451 C:A, **2831 C:A**, **2917 T:C**, **2978 G:A**, **3194 C:T**, **3437 G:A**, 3531 T:A, 3792 G:A, 3846 A:G, 4701 A:G, **4821 G:A**, 4881 G:T, 4959 A:T, 5016 T:C, 5148 G:A, 5562 A:G, 5679 A:G, 5733 A:G, 7524 T:C, 7725 T:A, 7962 A:G, 8461 T:C, 9408 T:C, **9851 G:A**, 9927 T:C, 10002 C:A, 10044 C:T, **10369 A:G**, 10407 T:C, **10831 A:G**, 10911 A:G, 10947 G:A, 11049 T:C, 11094 T:C, 11332 T:C | 14 | 439 H:D, 453 V:I, 554 V:M, 728 G:R, 767 P:L, 944 T:N, 973 Y:H, 993 R:K, 1065 S:L, 1146 R:Q, 1607 M:I, 3284 R:K, 3457 I:V, 3611 I:V |

Nonsynonymous nucleotide substitutions are bolded.

**Table S4.** Location and type of nucleotide and amino acid substitutions between AU526 and isolates from offspring born to dams infected with BVDV in early pregnancy.

| AU526:Offspring | Total | Location and type of nucleotide substitutions | Total | Location and type of amino acid substitutions |
| --- | --- | --- | --- | --- |
| AU526:B1A | 34 | 774 A:G, 783 C:T, 1008 T:C, **1315 C:G**, **1406 A:G**, **1660 G:A**, 1800 A:G, **1802 A:G**, **2055 A:G**, **2135 C:T**, **2211 A:T**, **2917 T:C**, **2978 G:A**, 3462 T:C, 4017 T:C, 4071 T:C, **4117 A:G**, 4701 A:G, 4881 G:T, 5094 T:C, 5562 A:G, 5733 A:G, 5862 G:A, 6702 T:C, **7105 G:A**, **7309 A:T**, 7362 T:C, 7524 T:C, 7575 C:T, 8079 T:C, **9851 G:A**, 10947 G:A, 11007 A:G, 11301 C:T | 13 | 439 H:D, 469 K:R, 554 V:M, 601 E:G, 685 I:M, 712 P:L, 737 E:D, 973 Y:H, 993 R:K, 1373 I:V, 2369 A:T, 2437 T:S, 3284 R:K |
| AU526:B2A | 26 | **175 G:A**, **437 A:G**, 549 G:A, 2208 T:C, **2246 C:T**, 2439 A:G, **2659 C:G^a^**, **2660 T:C^a^**, **3041 G:A**, 3292 C:T, **5613 C:A**, 5712 T:C, 6180 G:A, 6819 C:A, 7047 G:A, 7392 T:A, 8182 C:T, 8745 A:G, 8769 A:G, 9024 C:T, 9138 T:C, 9327 T:C, 10044 C:T, 10068 A:G, 10479 G:A, **10924 G:T** | 7 | 59 D:N, 146 K:R, 749 T:I, 887 L:A, 1014 G:E, 1871 D:E, 3642 A:S |
| AU526:B3A | 23 | **175 G:A**, **437 A:G**, 549 G:A, **1843 T:G**, 2208 T:C, 2439 A:G, **2659 C:G**, **2837 G:A**, 3292 C:T, **5613 C:A**, 5712 T:C, 6180 G:A, 6819 C:A, 7047 G:A, 7392 T:A, 8182 C:T, 8769 A:G, 9024 C:T, 9138 T:C, 9327 T:C, 10044 C:T, 10068 A:G, 10479 G:A | 6 | 59 D:N, 146 K:R, 615 L:V, 887 L:V, 946 R:Q, 1871 D:E |
| AU526:B4A | 28 | **175 G:A**, **437 A:G**, 549 G:A, **1654 T:C**, **2102 C:T**, **2135 C:T**, **2161 C:T^b^**, **2162 A:G^b^**, 2208 T:C, 2439 A:G, **2659 C:G**, **2837 G:A**, **3104 C:T**, 3292 C:T, **5613 C:A**, 5712 T:C, 6180 G:A, 6819 C:A, 7047 G:A, 7392 T:A, 8182 C:T, 8769 A:G, 9024 C:T, 9138 T:C, 9327 T:C, 10044 C:T, 10068 A:G, 10479 G:A | 10 | 59 D:N, 146 K:R, 552 S:P, 701 S:L, 712 P:L, 721 Q:W, 887 L:V, 946 R:Q, 1035 S:F, 1871 D:E |
| AU526:B5A | 25 | **175 G:A**, **437 A:G**, 549 G:A, 1689 A:G, **1828 G:A**, 2208 T:C, 2439 A:G, **2659 C:G**, **2837 G:A**, 3292 C:T, **5613 C:A**, 5712 T:C, 6180 G:A, 6819 C:A, 7047 G:A, 7122 A:G, 7392 T:A, 8182 C:T, 8769 A:G, 9024 C:T, 9138 T:C, 9327 T:C, 10044 C:T, 10068 A:G, 10479 G:A | 6 | 59 D:N, 146 K:R, 610 G:R, 887 L:V, 946 R:Q, 1871 D:E |
| AU526:B6A | 27 | **175 G:A**, **437 A:G**, 549 G:A, 2208 T:C, 2295 C:A, 2439 A:G, **2659 C:G**, **2837 G:A**, 3292 C:T, **5613 C:A**, 5712 T:C, 6180 G:A, **6301 C:T**, 6645 C:T, 6819 C:A, 7047 G:A, 7392 T:A, 8182 C:T, **8285 A:G**, 8769 A:G, 9024 C:T, 9138 T:C, 9327 T:C, 10044 C:T, 10068 A:G, 10479 G:A, 10647 G:A | 7 | 59 D:N, 146 K:R, 887 L:V, 946 R:Q, 1871 D:E, 2101 P:S, 2762 K:R |
| AU526:O1A | 49 | **53 C:T**, 327 A:T, 783 C:T, 1008 T:C, **1315 C:G**, **1330 G:T**, 1626 A:G, **1660 G:A**, **1738 T:A**, **1802 A:G**, 1833 A:G, **2211 A:T**, **2917 T:C**, **2978 G:A**, **3194 C:T**, **3206 T:A**, 3690 T:G, **4117 A:G**, **4261 A:G**, **4378 G:A**, 4701 A:G, 4881 G:T, 4899 A:T, 5148 G:A, 5553 A:G, 5562 A:G, 5679 A:G, 5733 A:G, 5811 T:C, 6114 G:T, 6216 A:T, 6645 C:T, 6858 T:C, 6996 A:G, **7105 G:A**, 7524 T:C, 7692 G:A, 8598 T:C, 8604 G:A, 9195 G:A, 9318 A:T, **9831 G:T**, **9851 G:A**, 10119 A:G, 10542 A:G, 10947 G:A, 11037 A:T, 11082 C:T, 11301 C:T | 17 | 18 S:F, 439 H:D, 444 V:L, 554 V:M, 580 S:T, 601 E:G, 737 E:D, 973 Y:H, 993 R:K, 1065 S:L, 1069 F:Y, 1373 I:V, 1421 T:A, 1460 E:K, 2369 A:T, 3277 K:N, 3284 R:K |
| AU526:O2A | 49 | 247 T:C, 669 A:G, 726 A:G, 783 C:T, 846 A:G, 978 T:C, 1008 T:C, 1254 A:G, **1315 C:G**, **1357 G:A**, 1446 A:G, 1626 A:G, **1660 G:A**, 1812 A:G, **2182 G:C**, 2451 C:A, **2644 G:A**, **2831 C:A**, **2917 T:C**, **2978 G:A**, **3194 C:T**, **3437 G:A**, 3531 T:A, 3792 G:A, 3846 A:G, 4701 A:G, **4821 G:A**, 4881 G:T, 4959 A:T, 5016 T:C, 5148 G:A, 5562 A:G, 5679 A:G, 5733 A:G, 7524 T:C, 7725 T:A, 7962 A:G, 8461 T:C, 9408 T:C, **9851 G:A**, 9927 T:C, 10002 C:A, 10044 C:T, 10407 T:C, **10831 A:G**, 10911 A:G, 10947 G:A, 11049 T:C, 11094 T:C | 13 | 439 H:D, 453 V:I, 554 V:M, 728 G:R, 882 V:M, 944 T:N, 973 Y:H, 993 R:K, 1065 S:L, 1146 R:Q, 1607 M:I, 3284 R:K, 3611 I:V |
| AU526:O2B | 51 | 247 T:C, **523 G:A**, 669 A:G, 726 A:G, 783 C:T, 846 A:G, 978 T:C, 1008 T:C, 1254 A:G, **1315 C:G**, **1357 G:A**, **1435 G:A**, 1446 A:G, 1626 A:G, **1660 G:A**, 1812 A:G, **2182 G:C**, 2451 C:A, **2831 C:A**, **2917 T:C**, **2978 G:A**, **3194 C:T**, **3437 G:A**, 3531 T:A, 3792 G:A, 3846 A:G, 4701 A:G, **4821 G:A**, 4881 G:T, 4959 A:T, 5016 T:C, 5148 G:A, 5562 A:G, 5679 A:G, 5733 A:G, 7524 T:C, 7725 T:A, 7962 A:G, 8461 T:C, 9408 T:C, **9851 G:A**, 9927 T:C, 10002 C:A, 10044 C:T, 10407 T:C, 10416 G:A, **10831 A:G**, 10911 A:G, 10947 G:A, 11049 T:C, 11094 T:C | 14 | 175 G:R, 439 H:D, 453 V:I, 479 G:R, 554 V:M, 728 G:R, 944 T:N, 973 Y:H, 993 R:K, 1065 S:L, 1146 R:Q, 1607 M:I, 3284 R:K, 3611 I:V |
| AU526:O4A | 52 | 247 T:C, 669 A:G, 726 A:G, 783 C:T, 846 A:G, 978 T:C, 1008 T:C, 1254 A:G, **1315 C:G**, **1357 G:A**, 1446 A:G, 1626 A:G, **1660 G:A**, 1812 A:G, **2182 G:C**, **2189 A:G**, 2451 C:A, **2648 C:T**, **2831 C:A**, **2917 T:C**, **2978 G:A**, **3194 C:T**, **3437 G:A**, 3531 T:A, 3792 G:A, 3846 A:G, 4701 A:G, **4821 G:A**, 4881 G:T, 4959 A:T, 5016 T:C, 5148 G:A, 5562 A:G, 5679 A:G, 5733 A:G, 7524 T:C, 7725 T:A, 7962 A:G, 8461 T:C, 9408 T:C, **9851 G:A**, 9927 T:C, 10002 C:A, 10044 C:T, 10137 A:G, **10369 A:G**, 10407 T:C, **10831 A:G**, 10911 A:G, 10947 G:A, 11049 T:C, 11094 T:C | 15 | 439 H:D, 453 V:I, 554 V:M, 728 G:R, 730 Q:R, 883 P:L, 944 T:N, 973 Y:H, 993 R:K, 1065 S:L, 1146 R:Q, 1607 M:I, 3284 R:K, 3457 I:V, 3611 I:V |

^a^ Nucleotide substitutions associated with the same amino acid substitution (887 L:A)

^b^ Nucleotide substitutions associated with the same amino acid substitution (721 Q:W)

Nonsynonymous nucleotide substitutions are bolded.

**Table S5.** Location and type of nucleotide and amino acid substitutions between BVDV-1b isolates from acutely infected dams and their offspring.

| Dam:Offspring | Total | Location and type of nucleotide substitutions | Total | Location and type of amino acid substitutions |
| --- | --- | --- | --- | --- |
| B1:B1A | 43 | 774 A:G, 783 C:T, 1008 T:C, 1017 T:C, **1315 C:G**, **1406 A:G**, **1660 G:A**, 1800 A:G, **1802 A:G**, **2055 A:G**, **2135 C:T**, **2211 A:T**, **2246 T:C**, **2917 T:C**, **2978 G:A**, 3462 T:C, **3763 C:T**, **3841 C:G**, 4017 T:C, 4071 T:C, **4117 A:G**, 4701 A:G, 4881 G:T, 5094 T:C, 5562 A:G, 5649 T:C, 5733 A:G, 5862 G:A, 5904 C:T, 6702 T:C, **7105 G:A**, **7120 A:G**, **7309 A:T**, 7362 T:C, 7524 T:C, 7575 C:T, 8079 T:C, 9138 C:T, **9851 G:A**, 10947 G:A, 11007 A:G, 11301 C:T, 11328 A:G | 17 | 439 H:D, 469 K:R, 554 V:M, 601 E:G, 685 I:M, 712 P:L, 737 E:D, 749 I:T, 973 Y:H, 993 R:K, 1255 L:F, 1281 P:A, 1373 I:V, 2369 A:T, 2374 K:E, 2437 T:S, 3284 R:K |
| B2:B2A | 38 | 133 T:C, **175 G:A**, **287 A:C**, **437 A:G**, 549 G:A, **1096 G:A**, **2078 C:T**, **2146 G:A**, 2208 T:C, 2439 A:G, **2659 C:G^a^**, **2660 T:C^a^**, **3041 G:A**, 3292 C:T, 4467 G:A, 5223 A:C, **5613 C:A**, 5712 T:C, 5733 G:A, 5982 A:G, 6180 G:A, 6534 A:C, 6819 C:A, 7047 G:A, **7120 A:G**, 7392 T:A, 7788 C:T, 8182 C:T, 8745 A:G, 8769 A:G, 9024 C:T, 9138 T:C, 9327 T:C, 9552 A:C, 10044 C:T, 10068 A:G, 10479 G:A, 10863 A:G | 10 | 59 D:N, 96 H:P, 146 K:R, 366 E:K, 693 S:F, 716 A:T, 887 L:A, 1014 G:E, 1871 D:E, 2374 K:E |
| B3:B3A | 4 | **1843 T:G**, 7416 C:A, **7559 A:G**, 7746 T:C | 2 | 615 L:V, 2520 K:R |
| B4:B4A | 10 | 912 A:G, **1654 T:C**, **1738 A:T**, **2102 C:T**, **2135 C:T**, **2161 C:T^b^**, **2162 A:G^b^**, 2562 T:C, **3104 C:T**, 6171 T:C | 6 | 552 S:P, 580 T:S, 701 S:L, 712 P:L, 721 Q:W, 1035 S:F |
| B5:B5A | 48 | **83 T:C**, **175 G:A**, **437 A:G**, 549 G:A, **975 A:C**, 1689 A:G, **1828 G:A**, 2208 T:C, 2439 A:G, 2475 G:A, **2659 C:G**, **2714 T:C**, 2739 T:C, 3292 C:T, **3494 A:G**, 3789 G:A, **5179 G:A**, **5613 C:A**, 5712 T:C, 6180 G:A, 6234 T:C, 6819 C:A, 7047 G:A, 7122 A:G, 7392 T:A, 7467 C:T, 7623 G:A, 7707 T:C, 8013 G:A, 8073 A:G, 8182 C:T, 8517 G:A, 8520 C:T, 8736 T:C, 8769 A:G, 9024 C:T, 9138 T:C, 9285 T:C, 9315 T:C, 9327 T:C, 9369 C:T, 10044 C:T, 10068 A:G, **10141 A:C**, 10455 A:G, 10479 G:A, 10683 C:T, 11577 A:G | 11 | 28 V:A, 59 D:N, 146 K:R, 325 Q:H, 610 G:R, 887 L:V, 905 F:S, 1165 K:R, 1727 A:T, 1871 D:E, 3381 N:H |
| B6:B6A | 7 | 2295 C:A, 5712 T:C, **6301 C:T**, 6645 C:T, 7392 T:A, **8285 A:G**, 10647 G:A | 2 | 2101 P:S, 2762 K:R |
| O1:O1A | 49 | **53 C:T**, 327 A:T, 783 C:T, 1008 T:C, **1315 C:G**, **1330 G:T**, 1626 A:G, **1660 G:A**, **1738 T:A**, **1802 A:G**, 1833 A:G, **2211 A:T**, **2917 T:C**, **2978 G:A**, **3194 C:T**, **3206 T:A**, 3690 T:G, **4117 A:G**, **4261 A:G**, **4378 G:A**, 4701 A:G, 4881 G:T, 4899 A:T, 5148 G:A, 5553 A:G, 5562 A:G, 5679 A:G, 5733 A:G, 5811 T:C, 6114 G:T, 6216 A:T, 6645 C:T, 6858 T:C, 6996 A:G, **7105 G:A**, 7524 T:C, 7692 G:A, 8598 T:C, 8604 G:A, 9195 G:A, 9318 A:T, **9831 G:T**, **9851 G:A**, 10119 A:G, 10542 A:G, 10947 G:A, 11037 A:T, 11082 C:T, 11301 C:T | 17 | 18 S:F, 439 H:D, 444 V:L, 554 V:M, 580 S:T, 601 E:G, 737 E:D, 973 Y:H, 993 R:K, 1065 S:L, 1069 F:Y, 1373 I:V, 1421 T:A, 1460 E:K, 2369 A:T, 3277 K:N, 3284 R:K |
| O2:O2A | 56 | 247 T:C, 669 A:G, 726 A:G, 846 A:G, 978 T:C, **988 A:G**, 1203 C:T, 1245 A:C, 1254 A:G, **1330 T:G**, **1357 G:A**, 1446 A:G, 1812 A:G, **1973 A:C**, **2182 G:C**, 2451 C:A, **2644 G:A**, **2831 C:A**, 3174 T:C, **3194 C:T**, **3437 G:A**, 3531 T:A, 3720 T:C, 3792 G:A, 3846 A:G, **3962 G:A**, **4261 G:A**, **4821 G:A**, 4956 T:C, 4959 A:T, 5016 T:C, 5181 A:G, 5679 A:G, 6009 G:A, 6372 G:A, 7725 T:A, 7836 G:A, 7962 A:G, 8451 C:A, 8461 T:C, **9034 A:G**, 9408 T:C, 9927 T:C, 10002 C:A, 10005 C:T, 10044 C:T, 10245 T:G, **10248 T:G**, 10407 T:C, **10831 A:G**, 10911 A:G, 11049 T:C, 11094 T:C, 11301 T:C, 11469 C:T, **11603 A:T** | 16 | 330 T:A, 444 L:V, 453 V:I, 658 N:T, 728 G:R, 882 V:M, 944 T:N, 1065 S:L, 1146 R:Q, 1321 R:K, 1421 A:T, 1607 M:I, 3012 T:A, 3416 D:E, 3611 I:V, 3868 E:V |
| O2:O2B | 58 | 247 T:C, **523 G:A**, 669 A:G, 726 A:G, 846 A:G, 978 T:C, **988 A:G**, 1203 C:T, 1245 A:C, 1254 A:G, **1330 T:G**, **1357 G:A**, **1435 G:A**, 1446 A:G, 1812 A:G, **1973 A:C**, **2182 G:C**, 2451 C:A, **2831 C:A**, 3174 T:C, **3194 C:T**, **3437 G:A**, 3531 T:A, 3720 T:C, 3792 G:A, 3846 A:G, **3962 G:A**, **4261 G:A**, **4821 G:A**, 4956 T:C, 4959 A:T, 5016 T:C, 5181 A:G, 5679 A:G, 6009 G:A, 6372 G:A, 7725 T:A, 7836 G:A, 7962 A:G, 8451 C:A, 8461 T:C, **9034 A:G**, 9408 T:C, 9927 T:C, 10002 C:A, 10005 C:T, 10044 C:T, 10245 T:G, **10248 T:G**, 10407 T:C, 10416 G:A, **10831 A:G**, 10911 A:G, 11049 T:C, 11094 T:C, 11301 T:C, 11469 C:T, **11603 A:T** | 17 | 175 G:R, 330 T:A, 444 L:V, 453 V:I, 479 G:R, 658 N:T, 728 G:R, 944 T:N, 1065 S:L, 1146 R:Q, 1321 R:K, 1421 A:T, 1607 M:I, 3012 T:A, 3416 D:E, 3611 I:V, 3868 E:V |
| O4:O4A | 3 | **2189 A:G**, **2648 C:T**, 10137 A:G | 2 | 730 Q:R, 883 P:L |

^a^ Nucleotide substitutions associated with the same amino acid substitution (887 L:A)

^b^ Nucleotide substitutions associated with the same amino acid substitution (721 Q:W)

Nonsynonymous nucleotide substitutions are bolded.

**Table S6.** Location and type of nucleotide and amino acid substitutions between BVDV-1b isolates obtained from persistently infected calves at different time points during the first six months of life.

| Offspring | Total | Location and type of nucleotide substitutions | Total | Location and type of amino acid substitutions |
| --- | --- | --- | --- | --- |
| B2A (84:168 doa) | 4 | **2246 T:C**, **3041 A:G**, 8745 G:A, **10924 T:G** | 3 | 749 I:T, 1014 E:G, 3642 S:A |
| B3A (birth:168 doa) | 3 | **1828 G:A**, **2648 C:T**, **2927 T:C** | 3 | 610 G :R, 883 P :L, 976 I :T |
| B4A (42:84 doa) | 6 | **1654 C:T**, **2102 T:C**, **2135 T:C**, **2161 T:C^a^**, **2162 G:A^a^**, **3104 T:C** | 5 | 552 P:S, 701 L:S, 712 L:P, 721 W:Q, 1035 F:S |
| B4A (42:168 doa) | 7 | **1654 C:T**, **2102 T:C**, **2135 T:C**, **2161 T:C^a^**, **2162 G:A^a^**, **2837 A:G**, **3104 T:C** | 6 | 552 P:S, 701 L:S, 712 L:P, 721 W:Q, 946 Q:R, 1035 F:S |
| B5A (42:84 doa) | 0 |  | 0 |  |
| B5A (42:168 doa) | 2 | **1828 A:G**, **2837 A:G** | 2 | 610 R:G, 946 Q:R |
| B6A (42:84 doa) | 0 |  | 0 |  |
| B6A (42:168 doa) | 0 |  | 0 |  |

^a^ Nucleotide substitutions associated with the same amino acid substitution (721 W:Q)

Nonsynonymous nucleotide substitutions are bolded.

doa, days of age

**Table S7.** Virus titers in passaged serum samples from acutely infected pregnant dams.

| Dam |  | Virus titers (TCID_50_/ml) | |
| --- | --- | --- | --- |
|  |  | 5 dpi | 7 dpi |
| B1 |  | NA | 6.2 × 10^4^ |
| B2 |  | NA | 2.0 × 10^6^ |
| B3 |  | NA | 6.2 × 10^5^ |
| B4 |  | 3.5 × 10^2^ | NA |
| B5 |  | 2.0 × 10^4^ | NA |
| B6 |  | NA | NA |
| O1 |  | 6.2 × 10^7^ | NA |
| O2 |  | 2.0 × 10^8^ | 2.0 × 10^7^ |
| O3 |  | NA | 2.0 × 10^7^ |
| O4 |  | NA | 6.2 × 10^6^ |
| O5 |  | 3.5 × 10^6^ | NA |
| O6 |  | NA | 2.0 × 10^7^ |

dpi, day postinoculation; NA, not applicable.

| Offspring |  | Virus titers (TCID_50_/ml) | | | |
| --- | --- | --- | --- | --- | --- |
|  |  | day of birth | 42 doa | 84 doa | 168 doa |
| B1A |  | 3.5 × 10^4^ | NA | NA | NA |
| B2A |  | NA | NA | 2.0 × 10^4^ | 2.0 × 10^5^ |
| B3A |  | 6.2 × 10^5^ | NA | NA | NA |
| B4A |  | NA | 3.5 × 10^5^ | 3.5 × 10^7^ | 3.5 × 10^7^ |
| B5A |  | NA | 6.2 × 10^6^ | 3.5 × 10^7^ | 3.5 × 10^7^ |
| B6A |  | NA | 3.5 × 10^2^ | 2.0 × 10^5^ | 1.1 × 10^5^ |
| O1A |  | 6.2 × 10^6^ | NA | NA | NA |
| O2A |  | 2.0 × 10^6^ | NA | NA | NA |
| O2B |  | 6.2 × 10^7^ | NA | NA | NA |
| O4A |  | 2.0 × 10^4^ | NA | NA | NA |

**Table S8.** Virus titers in passaged serum or tissue samples from offspring born to dams infected with BVDV.

doa, days of age; NA, not applicable.

**Table S9.** Results of qRT-PCR analyses of serum samples from acutely infected pregnant ewes.

| Dam |  | qRT-PCR^a^ | |
| --- | --- | --- | --- |
|  |  | 5 dpi | 7 dpi |
| O1 |  | + (400/275/36.2) | - (192/275/37.6) |
| O2 |  | + (363/311/28.5) | - (160/311/14.2) |
| O3 |  | - (7.3/281/0) | - (219/281/37.3) |
| O4 |  | - (4.5/380/0) | - (73.6/380/37.4) |
| O5 |  | - (289/434/24.5) | - (74.9/434/6.6) |
| O6 |  | - (338/503/14.6) | + (934/503/33.9) |

^a^ End relative fluorescence unit (RFU) values are given in parentheses followed by RFU cut-off and mean quantification cycle (Cq) values. dpi, day postinoculation; +, positive; -, negative.
